# *Azadirachta indica* (Neem) in Wound Healing: A Review of Bioactive Compounds, Mechanisms, and Current Translational Evidence

**DOI:** 10.3390/ijms27219591

**Published:** 2026-10-27

**Authors:** Rafa Alomari, Manal El-Zohri, Suzan Alharbi

**Affiliations:** 1 Department of Biological Sciences, Faculty of Science, King Abdulaziz University, Jeddah 21488, Saudi Arabia; ralomari0064@stu.kau.edu.sa (R.A.); mnzohri@aun.edu.eg (M.E.-Z.); 2 Department of Botany and Microbiology, Faculty of Science, Assiut University, Assiut 71516, Egypt

**Keywords:** *Azadirachta indica*, neem, wound healing, bioactive compounds, natural treatments, diabetic wound, burn wound, infected wound, incision and excision wounds

## Abstract

Chronic wounds are significantly increasing the risk of morbidity and amputation in diabetic patients and older people worldwide. Moreover, the widespread use of antibiotics has led to an increase in drug-resistant infections, complicating the healing process, prolonging hospitalization, and increasing the financial burden on healthcare systems. Consequently, plant-derived therapeutics have attracted considerable interest as accessible, biocompatible adjuncts in regenerative medicine. The neem plant (*Azadirachta indica*) is traditionally known as the “village pharmacy” due to its extensive pharmacological effects, such as antimicrobial, anti-inflammatory, antioxidant, and regenerative activities. This review covers the properties of different neem parts and extracts, their applications, and the molecular mechanisms of its essential bioactive compounds. In addition, it summarizes recent in vitro, in vivo, and emerging clinical findings across different wound types. Furthermore, it critically appraises key translational bottlenecks, including a primary technical barrier related to the lack of phytochemical standardization, non-linear dose responses, and the paucity of well-controlled human trials. Overall, while heterogeneous preclinical evidence supports the biological plausibility and wound-healing activity of *A. indica*, definitive clinical utility remains unproven across diverse patient cohorts. Future prospective methodology incorporating systematic chemical profiling and adequately powered randomized controlled trials will be essential to establish its clinical utility and safety profile in contemporary wound management.

## 1. Introduction

Cutaneous wound healing is a complex, multi-phase physiological process essential for restoring tissue integrity. Impaired healing, particularly in chronic, infected, or diabetic wounds, poses a significant global healthcare and economic burden. Conventional therapies often face critical limitations, including high costs, adverse effects, and the escalating crisis of antimicrobial resistance. This has prompted a strategic shift toward alternative phytotherapeutics and the integration of bioactive botanical agents into modern regenerative medicine.

Among these botanical candidates, the neem tree (*Azadirachta indica*)—often referred to as nature’s pharmacy—has emerged as a pivotal botanical agent [1]. Its multifaceted pharmacological and biological properties, including broad-spectrum antimicrobial, anti-inflammatory, antioxidant, and tissue-regenerative activities, are primarily attributed to key bioactive constituents such as nimbidin, azadirachtin, gedunin, and nimbin [2,3]. For centuries, various botanical organs of *A. indica* have been applied topically in traditional ethnomedicine to manage skin infections, thermal injuries, and chronic cutaneous wounds [4].

This review critically examines the therapeutic potential of *A. indica* for wound healing by bridging its historical applications with contemporary preclinical and translational evidence. Recent clinical investigations and pharmacological reviews highlight its growing applicability in modern therapeutic settings. These encompass dedicated initial trials on diabetic foot ulcers [5], comparative studies of polyherbal formulations for non-healing wounds [6], broad evaluations of its pharmacological potential in topical preparations [7], specific applications such as neem extract irrigation for diabetic ulcers [8], and observational reports in pediatric burn management [9]. While emerging studies in the broader literature also explore its use in dehisced surgical wounds and mixed-etiology cohorts, these translational findings underscore the profound complexities of clinical evaluation. Key challenges include isolating the specific efficacy of neem within polyherbal combinations and the overarching absence of standardized phytochemical dosing protocols. Therefore, rather than drawing definitive conclusions about its current clinical efficacy, this work provides a rigorous, etiology-specific analysis across diabetic, burn, infected, and surgical wound models. It synthesizes neem’s underlying molecular mechanisms—such as the modulation of inflammatory cascades and activation of angiogenic pathways—alongside emerging advanced biomaterial delivery platforms, while critically addressing the methodological heterogeneity that currently limits definitive evidence-based clinical translation.

### Literature Search Strategy and Selection Criteria

To provide a comprehensive and objective synthesis of the literature, a structured search was conducted across major electronic databases (PubMed, ScienceDirect, and Google Scholar) for peer-reviewed studies published from January 1992 to 30 June 2026. The search employed specific Boolean combinations of targeted keywords: (“*Azadirachta indica*” OR “neem”) AND (“wound healing” OR “wound closure” OR “tissue regeneration” OR “burns” OR “diabetic ulcer”) AND (“bioactive compounds” OR “extract” OR “phytotherapy”).

Following a multi-stage eligibility screening, primary empirical studies evaluating *A. indica* in cutaneous repair were identified and synthesized. Specific inclusion criteria encompassed in vitro assays, in vivo animal models, and human clinical evaluations that investigated crude extracts, fractions, or isolated phytochemicals. Studies evaluating multimodal polyherbal formulations were explicitly included, provided that *A. indica* constituted a defined, primary therapeutic component. This review is intentionally structured according to the therapeutic application of neem within each distinct wound etiology, categorizing interventions by formulation type to highlight their clinical relevance, current limitations, and future translational requirements.

## 2. Cutaneous Wound Pathology and the Clinical Burden

The skin constitutes the primary barrier against environmental pathogens, chemical insults, and mechanical trauma [10,11,12]. Cutaneous disruption results in acute or chronic wounds depending on the underlying physiological resolution [13,14]. While acute wounds (surgical incisions, trauma, and thermal burns) proceed through an orderly reparative cascade, they account for significant emergency utilization and economic burden [15,16]. In contrast, chronic wounds—predominantly diabetic foot ulcers (DFUs), venous leg ulcers, and pressure injuries—arrest in a persistent pathological state, failing to achieve anatomical integrity [15]. Driven by the global surge in diabetes, which affects an estimated 589 million individuals, non-healing wounds represent a major cause of global morbidity and substantial healthcare expenditure [16]. This burden is pronounced in high-prevalence regions; for instance, Saudi Arabia ranks among the highest globally in diabetic morbidity, driving heightened demand for accessible, bioactive regenerative agents [17,18].

## 3. Cellular Cascades, Therapeutic Frontiers, and Botanical Candidates

**Figure 1:**
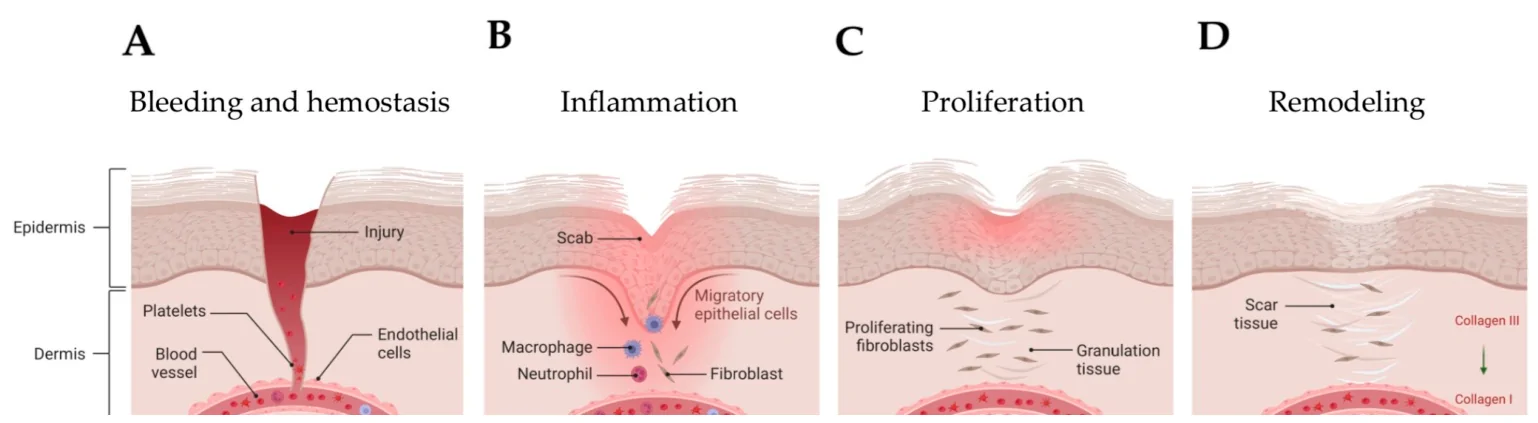
The physiological phases of cutaneous wound healing. (**A**). Bleeding and hemostasis: Vascular rupture, platelet plug aggregation, and fibrin clot formation. (**B**). Inflammation: Infiltration of neutrophils and macrophages to clear debris and phagocytose pathogens. (**C**). Proliferation: Granulation tissue development driven by proliferating fibroblasts, neovascularization, and migratory epithelial re-epithelialization. (**D**). Remodeling: Extracellular matrix maturation and scar tissue formation characterized by the replacement of collagen III with high-tensile collagen I. Created with BioRender.com.

Cutaneous tissue repair progresses through temporally overlapping, biologically coupled phases: hemostasis, inflammation, cellular proliferation, and extracellular matrix (ECM) remodeling (Figure 1).

Hemostasis and Inflammation (Figure 1A,B): Following vascular disruption, platelet aggregation and fibrin clot formation establish immediate hemostasis while triggering the release of chemotactic mediators, including PDGF and TGF-β (Figure 1A) [19,20]. Subsequent vasodilation permits the sequential infiltration of neutrophils and monocytes/macrophages to clear necrotic debris and bacterial contaminants via phagocytosis (Figure 1B) [19,21,22].

Proliferation (Figure 1C): Granulation tissue develops as activated fibroblasts deposit an immature ECM, while vascular endothelial growth factor (VEGF) drives angiogenic capillary sprouting (Figure 1C) [23,24]. Concurrently, migratory epithelial cells proliferate across the provisional matrix to reconstruct the epidermal barrier [19,20].

Remodeling (Figure 1D): The prolonged maturation phase is characterized by vascular regression and matrix metalloproteinase-mediated remodeling, wherein immature type III collagen is progressively substituted with structurally organized type I collagen bundles to restore tensile tissue integrity (Figure 1D) [23,25,26].

Persistent inflammatory states, microbial colonization, and vascular deficits severely arrest these phases, rendering traditional debridement and standard passive dressings insufficient [14,27,28]. While advanced modalities—such as bioengineered grafts, 3D bioprinting, and recombinant growth factors—have emerged, cost barriers, variable efficacy, and technical complexity limit their universal adoption [14,27,28]. Consequently, botanical therapeutics with multi-target pharmacological profiles (*Aloe vera*, *Centella asiatica*, and *Curcuma longa*) have attracted significant interest in their anti-inflammatory, antimicrobial, and pro-angiogenic activities [29,30]. However, incomplete phytochemical characterization and a primary technical barrier stemming from inadequate batch standardization, and scarce clinical trials remain major bottlenecks across herbal candidates, underscoring the necessity of rigorously evaluating potent, multi-mechanistic ethnobotanicals such as *Azadirachta indica* (neem) [30,31,32].

## 4. Neem (*Azadirachta indica*): Properties and Constituents

### 4.1. Neem Overview

**Figure 2:**
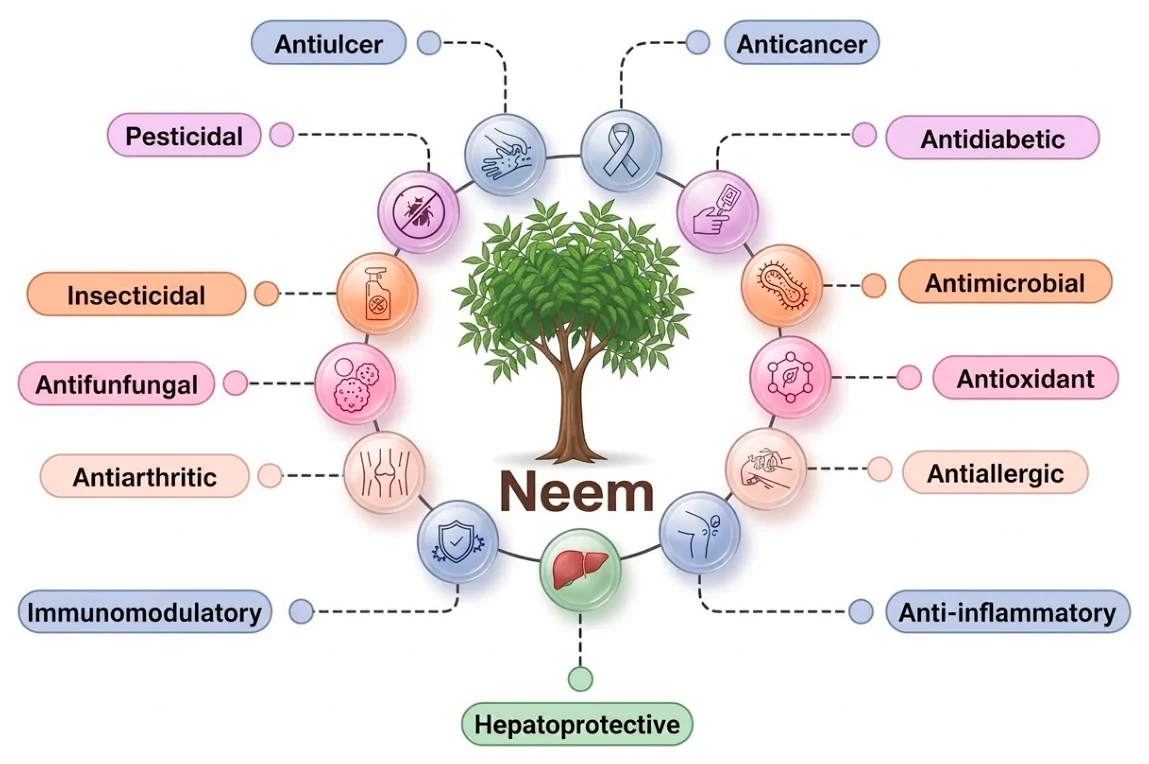
The therapeutic effects of the neem tree. Created using Canva (www.canva.com).

**Figure 3:**
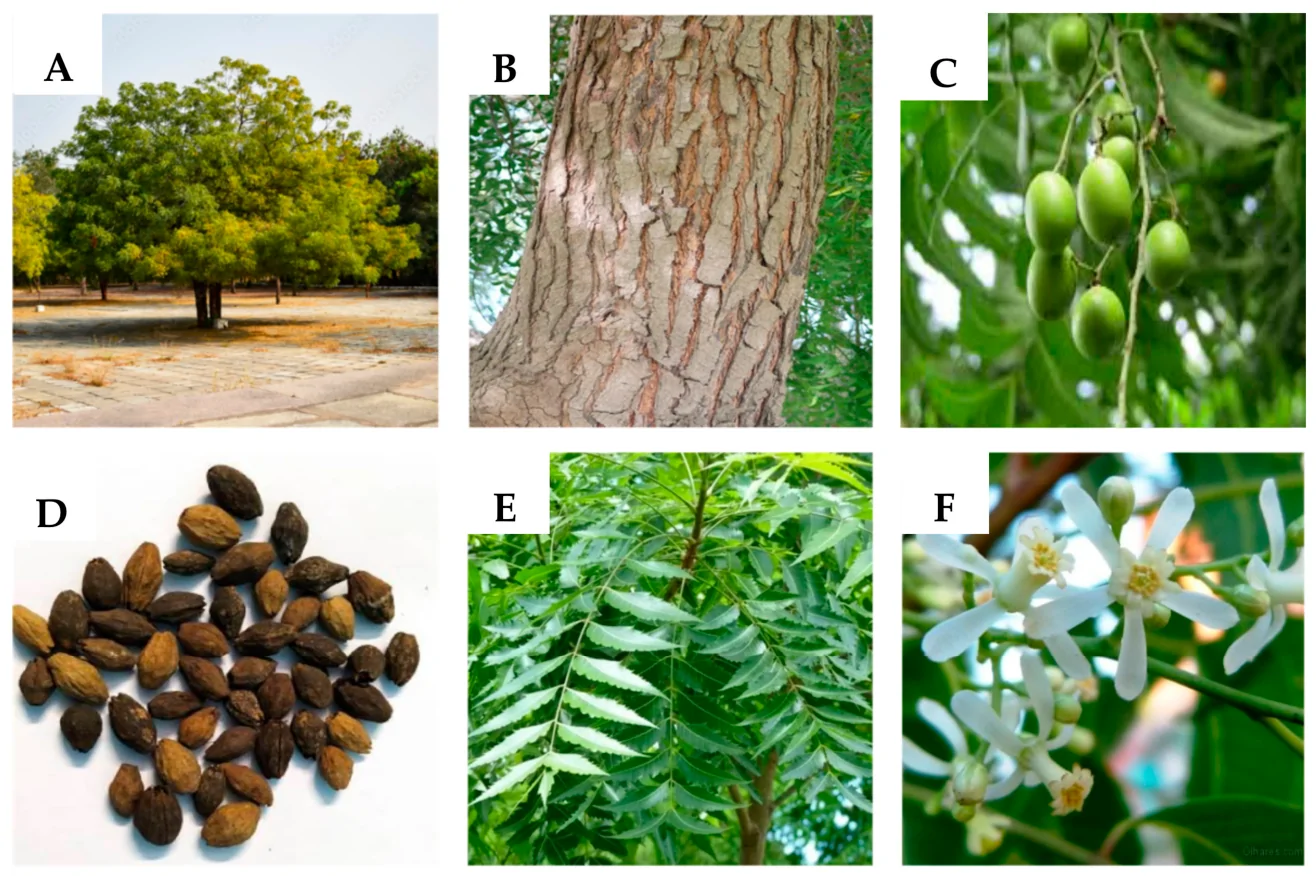
Neem and its different parts. (**A**). Tree. (**B**). Bark. (**C**). Fruits. (**D**). Seeds. (**E**). Leaves. (**F**). Flowers. Adapted from Kherde and Badhe [1], licensed under CC BY 4.0.

The scientific name of neem is *Azadirachta indica*, which belongs to the family Meliaceae. It possesses extensive medicinal properties (Figure 2) [1]. Neem has been used in South Asia for centuries in medicine, agriculture, and daily life (Figure 3A). It is widely cultivated across tropical and subtropical regions including South Asia and parts of Africa [33]. Neem trees are now widespread in Africa, the Caribbean, Central and South America, and other tropical regions. In Africa, they are well-established in over 30 countries, particularly in the Sahel. In the Americas, neem is found in southern Florida and across Central and South America [34]. For researchers, this widespread cultivation and naturalization imply that neem can be sourced with relative ease in these regions, either through local markets, agricultural extension services, or direct collection from naturalized stands.

It is extensively cultivated in dry and semi-dry regions because of its hardiness and adaptability [35]. It is a large tree that grows to 15–20 m tall, with straight, wide bark (Figure 3B). These trees usually require ample sunlight, which helps them tolerate high temperatures up to 49 °C. At the same time, it requires little water to grow and resists dry conditions. It starts fruiting after 3 to 5 years; it has oval and yellow-green fruits rich in oil that turn golden when ripe (Figure 3C). The fruit comprises three layers: a thin outer skin (exocarp), a fleshy middle layer (mesocarp), and an inner layer (endocarp) that contains the seed covered by a seed coat (Figure 3D). It has pinnately compound leaves with serrated leaflets (Figure 3E). Its flowers are arranged in an inflorescence, entirely white, containing approximately 150–200 flowers, measuring 5 mm in length and 8 mm in width (Figure 3F) [1].

Neem is used in traditional medical systems, including Ayurveda and Unani [36]. Antimicrobial and metabolic activities have been reported for neem preparations, but the strength of evidence varies by preparation and indication [33,37]. Traditional use and preclinical activity should therefore be distinguished from demonstrated clinical benefit.

Traditional applications include treatment of skin disorders and infections [4]. These applications provide a rationale for research, but do not by themselves establish efficacy or safety in acne, eczema, psoriasis, or viral skin disease.

### 4.2. Neem Bioactive Compounds

Neem contains over 300 bioactive compounds, distributed across its different parts. Table 1 shows the various parts of the neem tree, their essential active compounds, and their functions.

### 4.3. Mechanism of Action of Bioactive Compounds Related to Wound Healing

#### 4.3.1. Nimbidin

Nimbidin is a bitter tetranortriterpenoid mixture extracted from neem bark, leaves, and seed oil. It has demonstrated essential activities relevant to wound healing, such as anti-inflammatory, antimicrobial, and antifungal properties in neem extract studies [43]; however, in most wound-healing studies, these properties are inferred from the crude extract profile rather than demonstrated through direct quantification or isolation of nimbidin in the tested formulation. The anti-inflammatory activity of nimbidin has been examined directly in one isolation-level study using purified nimbidin [44]. Kaur et al. administered nimbidin (5–25 mg/kg orally) to rats and exposed rat peritoneal macrophages and human neutrophils to nimbidin in vitro. The study directly measured chemotaxis, phagocytosis, superoxide production, and the release of specific mediators, demonstrating that purified nimbidin inhibited macrophage migration to the peritoneal cavity in response to inflammatory stimuli and suppressed macrophage production of nitric oxide (NO) and prostaglandin E_2_ (PGE_2_) in LPS-stimulated cells. The mechanism of NO suppression was traced experimentally to reduced induction of inducible nitric oxide synthase (iNOS), without direct catalytic inhibition of the enzyme [44]. In the same study, nimbidin also directly attenuated degranulation in stimulated human neutrophils, measurably reducing the release of β-glucuronidase, myeloperoxidase (MPO), and lysozyme [44]. These findings represent experimentally demonstrated mechanisms specific to isolated nimbidin, not to crude neem extract.

Singh et al. evaluated cold-pressed neem oil in chronic wounds and measured angiogenesis and tissue DNA content [6]. Because the oil was not characterized by quantitative nimbidin analysis, these clinical observations cannot establish a nimbidin-specific effect. Likewise, antimicrobial activity attributed to neem preparations should not be assigned to nimbidin without constituent-specific experiments. The leukocyte findings reported by Kaur et al. support an anti-inflammatory action of the tested nimbidin preparation, but do not demonstrate accelerated cutaneous wound closure [44].

#### 4.3.2. Azadirachtin

**Figure 4:**
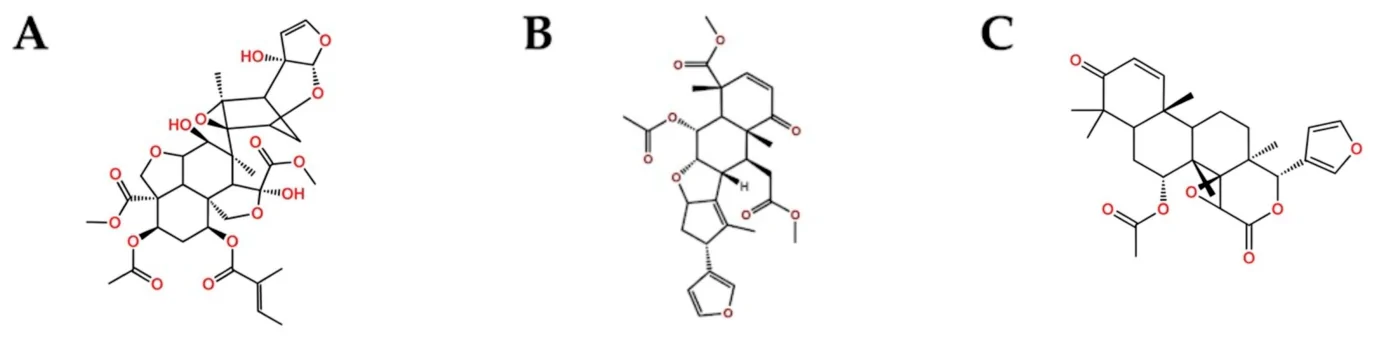
Neem bioactive compounds contributing to wound healing. (**A**). Azadirachtin. (**B**). Nimbin. (**C**). Gedunin. Created with Molview “https://app.molview.com (accessed on 15 March 2025)”.

Azadirachtin is a predominant bioactive compound derived from the seeds of neem (Figure 4A). It is one of four principal limonoids (Azadirachtin, Salannin, Meliantriol, and Nimbin) characterized by insecticidal and pesticidal activity [2]; this classification is drawn from a narrative review and reflects a consensus from prior literature rather than original experimental analysis [2]. Although conventionally identified as a biopesticide, its experimentally demonstrated anti-inflammatory and angiogenic properties are highly relevant to wound healing [45]. Antimicrobial activity has additionally been attributed to azadirachtin based on prior literature, though the supporting data derive from crude neem extract studies rather than trials with isolated azadirachtin. The antimicrobial activity reported for neem preparations helps prevent wound infection by inhibiting the growth of bacteria such as *Staphylococcus aureus* and *Pseudomonas aeruginosa* [46]; however, these findings were obtained with crude leaf extracts and have not been replicated with isolated azadirachtin, so the attribution of this activity specifically to azadirachtin remains an inference from the broader neem literature. The claim that azadirachtin reduces cellular swelling and tissue damage is similarly inferred and has not been directly demonstrated in wound-specific experimental models.

In contrast, the anti-inflammatory and angiogenic mechanisms of azadirachtin have been directly examined using a purified compound. He et al. (2020) administered highly purified azadirachtin A (≥95% purity, 100–200 mg/kg/day orally) to rats in a random skin flap model and directly measured inflammatory and angiogenic endpoints [45]. The study demonstrated that azadirachtin A significantly reduced serum levels of TNF-α and IL-6, measured by ELISA, and decreased tissue expression of IL-1β and IL-6 by immunohistochemistry, while concurrently increasing the expression of VEGF, thereby enhancing blood vessel formation at the wound site (Table 2). These cytokine and growth factor findings represent experimentally demonstrated mechanisms specific to purified azadirachtin A in an in vivo model. While upregulated VEGF supports nutrient delivery during early tissue repair, direct acceleration of granulation tissue or cell proliferation remains unverified in excisional wound models [45].

#### 4.3.3. Nimbin

Nimbin (a tetranortriterpenoid) is a bioactive compound that can be obtained from neem leaves and seeds (Figure 4B) [36]. Based on historical records compiled in prior reviews, nimbin was first isolated as a bitter compound from neem oil in 1942 [37]. The compound exhibits various biological activities that support wound healing, including antimicrobial, anti-inflammatory, antioxidant, and antifungal properties [33]; these properties are from prior literature reviews and have not been uniformly demonstrated for isolated nimbin in direct wound-healing experiments. Anti-inflammatory agents like nimbin can help balance the inflammatory response and enhance the proliferative phase in wound healing. In direct experimental work using purified Nimbin (N1) isolated from neem seed kernels alongside semi-synthetic analogs N2 and N3, Sudhakaran et al. demonstrated anti-inflammatory activity through in vitro lipoxygenase inhibition (64.1% at 25 μM for N1), proteinase inhibition (65.6%), and red blood cell membrane stabilization assays in human blood. These represent experimentally demonstrated mechanisms for isolated nimbin. However, the reduction in pro-inflammatory cytokines such as TNF-α and IL-1β or the direct modulation of iNOS, COX-2, and Nuclear factor kappa B (NF-κB) nuclear translocation was not directly measured in this study; the attribution of cytokine-level and transcriptional effects to nimbin in the original text is not supported by the cited reference and instead reflects inferences drawn from prior studies of crude neem preparations [47]. In addition, the same study directly demonstrated that purified nimbin can regulate reactive oxygen species (ROS), reducing intracellular ROS to 63.65% of control levels in a zebrafish (*Danio rerio*) in vivo model using the DCF-DA assay, and attenuating lipid peroxidation to 66% of control, supporting antioxidant activity under the tested conditions (Table 2).

Recent studies have found that neem leaf extract, which primarily contains azadirachtin and nimbin, exhibits directly measured strong antioxidant activity (60.03% DPPH radical inhibition at 1 mg/mL) [48]; however, nimbin was not isolated in this formulation, and this antioxidant finding reflects the activity of the crude extract rather than the specific contribution of nimbin [48]. Furthermore, prior literature reviews report that neem has antimicrobial activity against *Staphylococcus aureus*, *Escherichia coli*, and *Pseudomonas aeruginosa*, which cause wound infections [37]; direct experimental evidence for this activity using isolated nimbin in wound infection models is not provided in the cited reference. Collectively, the available evidence supports experimentally demonstrated roles for purified nimbin in suppressing inflammatory enzyme activity and attenuating oxidative stress in a zebrafish in vivo model. By contrast, the specific reduction in pro-inflammatory cytokines and antimicrobial effects against wound pathogens attributed to nimbin in prior accounts are drawn from crude neem extract literature and have not been directly established for isolated nimbin in wound-healing contexts. Consequently, while these findings substantiate the biological activities of isolated nimbin, its translational potential in mammalian wound models remains unconfirmed.

#### 4.3.4. Gedunin

**Figure 5:**
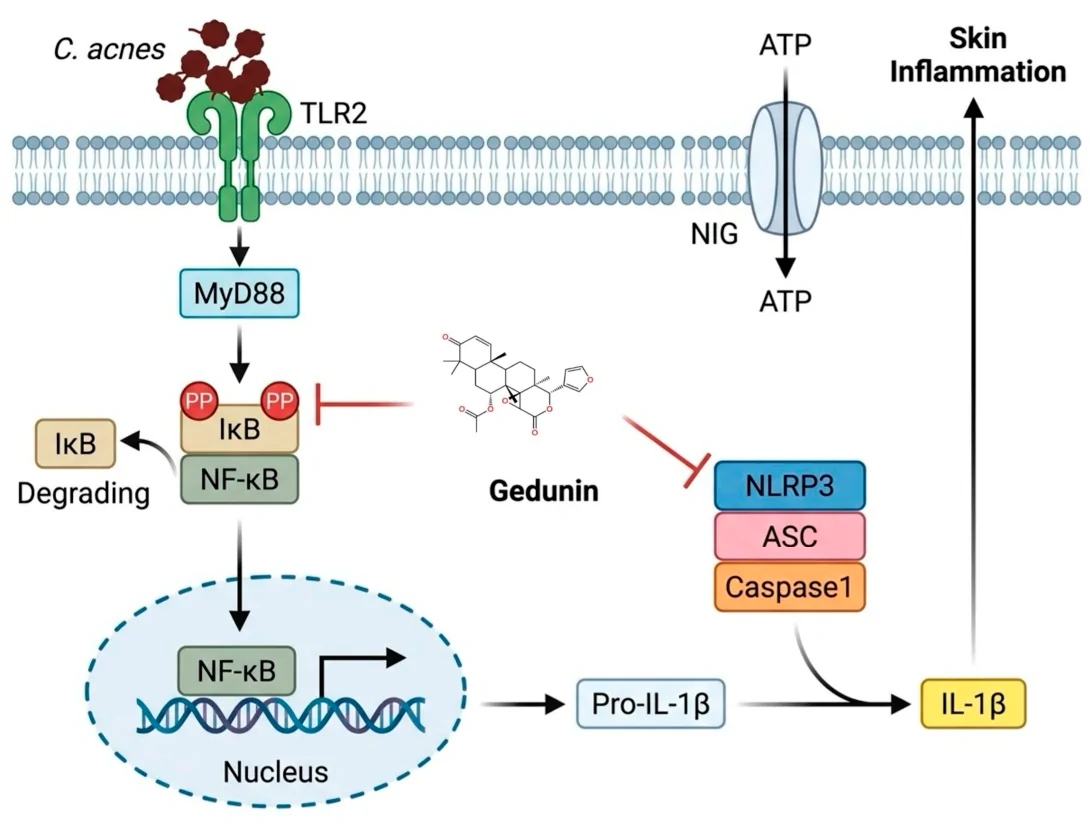
Gedunin attenuates *Cutibacterium acnes*-induced inflammation by modulating the TLR2/NF-κB and NLRP3 inflammasome signaling pathways. Binding of *C. acnes* to Toll-like receptor 2 (TLR2) recruits MyD88 to trigger downstream activation of the NF-κB cascade. Rather than directly blocking IκB kinase (IKK), gedunin mediates the attenuation of TLR2/NF-κB signaling, suppressing the phosphorylation and subsequent degradation of IκB. This keeps the NF-κB transcription factor sequestered in the cytoplasm and limits its nuclear translocation, thereby downregulating the gene transcription of pro-inflammatory mediators, including pro-IL-1β. Simultaneously, gedunin suppresses the assembly of the NLRP3 inflammasome complex (NLRP3, ASC, and caspase-1) stimulated by nigericin (NIG) and adenosine triphosphate (ATP), thereby inhibiting caspase-1-dependent cleavage of pro-IL-1β into mature IL-1β. Consequently, this dual inhibitory action significantly reduces IL-1β secretion and mitigates skin inflammation. Adapted from Sim et al. [49] licensed under CC BY 4.0 “http://creativecommons.org/licenses/by/4.0/ (accessed by 29 August 2026)”.

Gedunin is a bioactive limonoid mainly found in neem seeds (Figure 4C). It is known for its anti-inflammatory and anti-allergic properties, which suppress allergic features such as edema formation, allergic eosinophilia, and vascular permeability responses, and control allergic diseases [49,50]. It suppresses the NF-κB signaling pathway triggered by certain inflammatory mediators. NF-κB activation begins when inflammatory stimuli such as TNF-α, IL-1α, or bacterial components like *Cutibacterium acnes* interact with Toll-like receptors (TLRs) or tumor necrosis factor receptors (TNFRs). This interaction recruits myeloid differentiation primary response 88 (MyD88), an adaptor protein that activates a signaling cascade leading to activation of the IκB Kinase Complex [49,51]. Then, NF-κB translocates to the nucleus and binds to DNA to promote transcription of pro-inflammatory genes, including IL-1β, TNF-α, and COX-2. Gedunin interferes with this pathway by promoting the attenuation of TLR2/NF-κB signaling and suppressing IκB phosphorylation and degradation, thereby blocking NF-κB nuclear translocation and keeping it sequestered in the cytoplasm (Figure 5). Furthermore, gedunin exerts additional anti-inflammatory effects by inhibiting NOD-like receptor protein 3 NLRP3 inflammasome assembly, thereby reducing caspase-1 activation and the maturation of pro-IL-1β [49,51].

Additionally, gedunin suppresses MyD88/TLR2 signaling, reducing the activation of downstream kinases required for NF-κB activation [49,51]. By limiting these inflammatory pathways, gedunin may reduce excessive inflammation and support the cellular environment required for tissue repair. In normal human dermal fibroblasts, gedunin increased collagen production by up to 136.8%, creating a favorable environment for wound healing and tissue regeneration [49,50]. However, because these cellular actions and matrix-inductive properties were demonstrated in cultured human cells and cutaneous inflammatory models rather than in mammalian excisional or incisional wound models, their capacity to drive accelerated wound closure in clinical settings remains to be confirmed (Table 2).

A comparative synthesis of the direct molecular endpoints, phenotypic wound outcomes, and levels of experimental evidence for these isolated bioactives is presented in Table 2.

**Table 2:** Mechanistic evidence and molecular endpoints of characterized *Azadirachta indica* bioactive constituents in wound healing.

Compound	Purity/Preparation	Experimental Model	Direct Molecular Endpoint	Inferred/Postulated Wound Phenotype	Evidence Classification
**Nimbidin** [44]	Purified isolate (tetranortriterpenoid fraction)	In vitro (rat peritoneal macrophages & human neutrophils); in vivo acute inflammation	Suppresses macrophage chemotaxis, phagocytosis, NO production (via attenuated iNOS induction), and PGE_2_ release; attenuates neutrophil degranulation (MPO, β-glucuronidase, lysozyme)	Attenuation of excessive wound inflammation; hypothesized transition to the proliferative phase (unverified in cutaneous models)	Directly demonstrated for anti-inflammatory mediator suppression in leukocytes; wound-healing phenotype is inferred
**Azadirachtin** [45]	Isolated pure standard (purity ≥ 95%, isomer A)	In vivo rat ischemic wound/random skin flap model	Upregulates VEGF and CD31 expression; promotes eNOS phosphorylation; downregulates caspase-3, IL-1β, and TNF-α.	Accelerated neovascularization, elevated microvessel density, and reduced tissue necrosis in ischemic tissue.	Directly demonstrated in cutaneous flap model
**Nimbin** [47]	Pure isolate (N1) and semi-synthetic analogs	In vitro enzyme/membrane assays & in vivo zebrafish (*Danio rerio*) oxidative stress model	Inhibits lipoxygenase (LOX) and proteinase; stabilizes red blood cell membranes; attenuates intracellular ROS generation and lipid peroxidation.	Hypothesized mitigation of oxidative tissue stress (unvalidated in mammalian cutaneous wound models)	Directly demonstrated for in vitro enzymatic/membrane stabilization and in vivo antioxidant actions in zebrafish; wound-healing phenotype is inferred
**Gedunin** [49,50]	Pure limonoid isolate	In vitro human keratinocytes, fibroblasts, and cutaneous inflammatory/acne model	Inhibits IκBα degradation and phosphorylation; suppresses NF-κB p65 nuclear translocation; reduces pro-inflammatory mediators; upregulates collagen production in human dermal fibroblasts	Mitigation of cutaneous inflammatory stress; hypothesized matrix synthesis enhancement (unvalidated in mammalian excisional models)	Directly demonstrated for in vitro anti-inflammatory signaling and collagen upregulation; in vivo wound-healing phenotype is inferred

### 4.4. Plant Parts Used in Wound Healing and Their Extraction

Different parts of the neem tree have been studied in wound healing due to their medicinal properties. Its various parts are rich in bioactive compounds collectively called triterpenoids or limonoids. These compounds help with wound healing through various therapeutic activities, such as antibacterial, anti-inflammatory, and antioxidant activities [2]. These bioactive compounds can prevent infections, stimulate collagen synthesis, and enhance re-epithelialization and angiogenesis [2,3]. Neem is commonly used as an extract in wound irrigation, dressings, and other topical preparations [7,8]. Ethanol extraction is the most common method for extracting bioactive compounds from neem leaves and seeds, which helps clear the wound of bacteria and reduces healing time [3,7]. In addition, neem bark is also used in wound healing due to its antimicrobial and anti-inflammatory properties. For instance, Sahni and Dubey combined topical neem leaf decoction with an oral powder containing leaves, stem and root bark, flowers, and fruits [52]. Moreover, neem seed oil is rich in fatty acids and bioactive compounds that accelerate healing. Because it helps the skin retain moisture and maintain a soft texture during the healing process, it is produced by cold pressing and solvent extraction and then emulsified for use in wound dressings [3]. Overall, the different parts of neem have been extensively studied using various extraction methods due to their therapeutic properties in wound healing.

### 4.5. Neem and Different Types of Wounds

#### 4.5.1. Diabetic Wounds

Diabetic wounds are a complication of diabetes mellitus caused by uncontrolled high blood sugar levels, leading to nerve damage (neuropathy) and peripheral arterial disease. The healing process is often delayed because of prolonged inflammation, reduced oxygen supply, and an increased risk of infection. Without proper treatment, these wounds can lead to severe complications such as infections, tissue death (gangrene), and amputations [53]. The neem tree has shown significant potential in healing diabetic wounds due to its wound-healing, anti-inflammatory, and antimicrobial properties, as evidenced by both clinical and preclinical studies.

Clinical evaluation of neem in diabetic foot ulcers (DFUs) is currently derived from two independent patient cohorts represented across three publications [5,6,8]. In the first and only dedicated clinical trial, Jayalakshmi and colleagues initially published preliminary findings [8] followed by a full report of a quasi-experimental repeated-measures clinical trial [5]. This trial evaluated a single patient cohort that initially enrolled 200 patients with Wagner Grade I–III diabetic foot ulcers (DFUs), of whom 160 completed the 4-week protocol and were analyzed (*n* = 80 per group; 27 patients lost to follow-up and 13 excluded due to incomplete assessment) [5]. Baseline wound characteristics demonstrated initial comparability in wound area (*p* = 0.318), wound duration (*p* = 0.912), and HbA_1_c status (*p* = 0.772), although significant baseline disparities existed between cohorts in Wagner ulcer grading (*p* = 0.001), tissue bed composition (*p* = 0.013), and baseline exudate levels (*p* < 0.001). Topical irrigation with freshly prepared aqueous neem leaf extract (10 mL delivered via a 19-gauge needle twice weekly for 4 weeks) demonstrated marked therapeutic superiority over normal saline control. Specifically, neem irrigation reduced absolute mean wound surface area from 17.14 ± 21.45 cm^2^ at baseline to 7.04 ± 16.72 cm^2^ at week 4 (*p* < 0.001), representing a 58.9% relative reduction in mean wound area, compared with a reduction from 19.84 ± 23.97 cm^2^ to 14.31 ± 21.43 cm^2^ in the saline cohort (*p* < 0.03; 27.9% reduction) [5]. Furthermore, Pressure Ulcer Scale for Healing (PUSH) total scores improved significantly in the neem group (decreasing from 11.975 to 6.704) compared to saline (12.937 to 9.153), yielding a statistically significant difference in mean score reduction between groups (mean difference = 2.449, t = 4.457, *p* < 0.001) [5]. In evaluable responder analyses at week 4, 82.3% (51/62) of patients in the neem group achieved ≥ 50% percentage wound area reduction (PWAR) compared to 55.8% (29/52) in the saline arm (χ^2^-derived *p* = 0.002) [5]. Regarding safety and tolerability, no systemic complications, adverse events, or significant alterations in vital signs or clinical and physiological parameters (body temperature, blood pressure, pulse rate, respiration, or pain scores; *p* > 0.05) were observed, alongside a non-significant trend toward reduced clinical infection grade (*p* = 0.053) [5]. While these quantitative findings provide preliminary clinical evidence, the study benchmarked neem solely against normal saline rather than contemporary standard-of-care dressings (e.g., hydrocolloids or active enzymatic debriding agents), which limits contextualization within modern wound management pathways.

**Figure 6:**
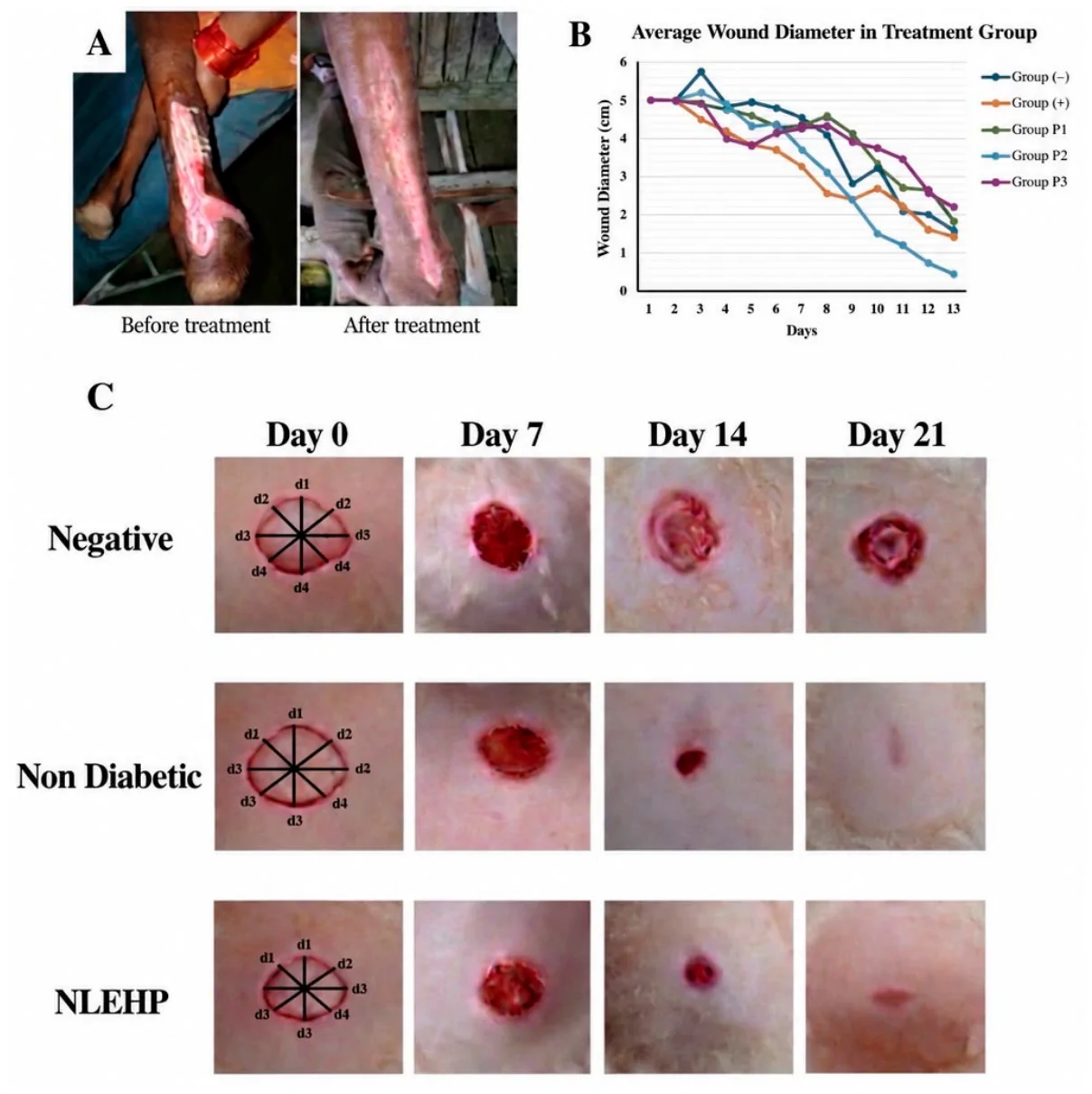
The therapeutic efficacy and histopathological impact of *Azadirachta indica* in diabetic wound healing. (**A**). Clinical photographs showing progressive ulcer area reduction and re-epithelialization in a chronic lower-extremity diabetic ulcer after 8 weeks of combined topical cold-pressed neem seed oil and oral turmeric (Haridra) therapy (representative case). Reproduced from Singh et al. [6], open-access under CC BY-NC-SA license. (**B**). In vivo quantitative wound contraction profile illustrating average excision wound diameters (mm) over a 13-day period in an STZ-induced diabetic rat model across control, povidone-iodine, and virgin coconut oil (VCO) formulations containing 5% (P1), 10% (P2), and 15% (P3) neem leaf extract. Adapted from Dewi et al. [54], licensed under CC BY 4.0. (**C**). Macroscopic photographic progression of full-thickness excisional cutaneous wound closure in diabetic Wistar rats treated with neem leaf extract hydrogel patches (NLEHP) compared to untreated diabetic (Negative) and healthy (Non-Diabetic) controls evaluated across days 0, 7, 14, and 21. Reproduced from Lestari et al. [55], licensed under CC BY 4.0.

When combined with turmeric (Haridra), neem oil produced the best healing outcomes for chronic diabetic wounds—a clinical study enrolling 60 patients allocated across three arms: Group I (topical cold-pressed neem seed oil, *n* = 16), Group II (oral Haridra powder capsules, 1 g three times daily, *n* = 11), and Group III (combined topical neem oil and oral Haridra, *n* = 20) [6]. Across 47 protocol-compliant patients presenting with heterogeneous chronic ulcers (of which only a subgroup of 20 patients [42.55%] had confirmed diabetic lower extremity ulcers), wounds were monitored at two-week intervals until complete re-epithelialization, with 50% healing evaluated at weeks 4, 8, and 12 (Figure 6A) [6]. At 8 weeks of intervention, ≥50% wound healing was documented in 70.0% of patients receiving combination therapy (Group III), compared to 43.8% in the neem monotherapy arm (Group I) and 18.2% in the turmeric monotherapy arm (Group II) (*p* = 0.02) [6]. In the specific diabetic ulcer cohort at 8 weeks, ≥50% healing was achieved in 75% of patients in Group III versus 40% in Group I and 0% in Group II (*p* < 0.05) [6]. Histopathological wound biopsies at 4 weeks demonstrated significant microvascular induction, with Microscopic Angiogenesis Grading System (MAGS) scores increasing to 43.75 ± 12.8 in Group III compared to 39.56 ± 13.91 in Group I and 29.91 ± 8.21 in Group II (*p* = 0.02 post-treatment) [6]. Similarly, tissue (DNA) concentrations rose significantly in the combination arm to 205.28 ± 115.78 ng/μL compared to 161.93 ± 70.15 ng/μL in Group I and 106.55 ± 76.92 ng/μL in Group II (*p* = 0.023) [6]. No adverse effects were reported across the trial cohort [6]. However, the inclusion of mixed wound etiologies, the small sample size of the diabetic subgroup, and the absence of intention-to-treat (ITT) modeling for the 13 dropouts warrant cautious clinical interpretation.

Similarly, Dewi et al. [54] evaluated a concentration-dependent topical formulation of neem leaf extract (5%, 10%, and 15%) incorporated into virgin coconut oil (VCO) in an STZ-induced diabetic rat excision wound model. Male white rats (2–3 months old, 100–120 g; *n* = 5/group, total *n* = 25) received a 5 mm excision wound after diabetes induction, and wounds were treated daily for 14 days. The study included a negative control (0.9% NaCl), a positive control (povidone-iodine), and three neem treatment groups. The 10% neem leaf extract formulation produced the greatest reduction in serum IL-6 levels and promoted faster wound closure compared with the lower and higher concentrations, while the 15% formulation did not provide additional benefit (Figure 6B) [54]. This non-linear response indicates that a higher nominal extract concentration did not necessarily improve the measured outcomes. Cytotoxicity, vehicle effects, and other explanations require direct testing.

Modern delivery systems have further advanced the application of neem in diabetic wound care, though direct comparisons between formulations remain challenging. A hydrogel loaded with neem leaf extract [male Wistar rats; *n* = 5/group, total *n* = 15; diabetes induced by IP injection of alloxan at 150 mg/kg; 1.5 cm diameter full-depth excision wound; hydrogel patch replaced every 3 days; assessed on days 0, 7, 14, and 21] achieved wound closure comparable to non-diabetic healing (*p* > 0.05) over 21 days (Figure 6C) [55]. These findings suggest that the hydrogel matrix may facilitate the sustained delivery of active phytochemicals within the diabetic wound bed, though clinical extrapolation remains to be confirmed.

In contrast, neem-based silver nanoparticles (AI-AgNPs) achieved 97.87% wound contraction within 20 days in diabetic mice [male Swiss albino mice, 20 weeks old, weighing 28–32 g; *n* = 3/group, total *n* = 15; diabetes induced by IP injection of STZ at 150 mg/kg; thermal wound model employing a 10% deep partial-thickness injury induced at 70 °C for 35 s—rather than a standard excision model; nanoparticle formulations (2.5 and 5 mg AgNPs) in a silver sulfadiazine (SSD) ointment base; daily dressing changes; wounds assessed at days 5, 10, 15, 20, and 25], with documented promotion of angiogenesis and tissue regeneration [56].

Reinforcing electrospun delivery in diabetic environments, Rajora et al. engineered a nanofibrous scaffold composed of *Azadirachta indica* gum polysaccharide (AIG, 3% *w*/*v*), polyvinyl alcohol (PVA, 7% *w*/*v*), and curcumin (CUR, 40 mg/10 mL). The resulting uniform non-woven nanofibers (diameter: 440 ± 80 nm; tensile strength: 3.56 MPa; entrapment efficiency: 87.40%) exhibited diffusion-controlled biphasic release kinetics. In an STZ-induced diabetic mouse excision wound model (*n* = 6/group; 8 mm full-thickness dorsal wound), the CUR-loaded AIG nanofiber mat accelerated healing, achieving complete (100%) wound contraction by day 18 (95% CI: 99.85–100; *p* = 0.0004) compared to 98.13% in commercial antibiotic ointment and 81.83% in untreated diabetic controls. Importantly, histopathological evaluation via hematoxylin and eosin (H&E) staining provided direct cellular evidence of tissue regeneration: treated wounds demonstrated thick, continuous re-epithelialization, high fibroblast proliferation, extensive neovascularization, and densely organized collagen bundles, which correlated with elevated hydroxyproline deposition (5.1 μg/mg dry tissue vs. 1.8 μg/mg in untreated diabetic controls) [57].

Although these formulations demonstrate promising efficacy, cross-study comparisons are not straightforward: studies utilized varied animal models (rats vs. mice), distinct wound induction modalities, heterogeneous outcome metrics, and divergent observation windows. These methodological disparities prevent definitive conclusions about which delivery platform is superior and highlight a critical gap in the literature—the absence of standardized experimental protocols for preclinical diabetic wound studies.

Several important knowledge gaps remain unaddressed. First, the vast majority of evidence is derived from animal models, and only one clinical trial specifically dedicated to exclusive DFU cohorts has been identified⁠, which substantially limits the translational validity of the available findings. Second, the plant parts used (leaves vs. oil vs. bark vs. gum), extraction solvents (aqueous, ethanolic, methanolic), and extract concentrations vary considerably across studies, making it difficult to establish reproducible therapeutic parameters or structure–activity relationships. Since neem oil is predominantly rich in fatty acids and triterpenoids (e.g., azadirachtin, nimbin), while leaf extracts are enriched in flavonoids (e.g., quercetin) and polyphenols, these compositional differences may partially explain inconsistencies in outcomes across studies. Third, comparator selection across the reviewed studies was inconsistent. Although some studies used established agents such as povidone-iodine, commercial antibiotic ointments, or silver sulfadiazine, standardized comparisons with contemporary advanced wound-care interventions remain limited. Finally, the long-term safety profile of neem preparations in immunocompromised diabetic patients—particularly regarding sensitization, nephrotoxicity from systemic absorption, or interactions with antidiabetic medications—is insufficiently characterized in the reviewed studies. Addressing these gaps through well-designed, randomized controlled trials using standardized neem preparations is essential before evidence-based clinical recommendations can be established. Methodological characteristics and key limitations of all reviewed studies are summarized in Supplementary Table S1.

#### 4.5.2. Burn Wounds

Burn wounds are injuries caused by extreme temperatures, chemicals, electricity, friction, or radiation. They are classified by severity as first-degree (superficial), second-degree (partial-thickness), third-degree (full-thickness), and fourth-degree burns, which damage deeper tissues such as muscle and bone [58]. Neem has been investigated as a therapeutic agent for burn wounds through preclinical and limited clinical studies, with findings generally supporting its anti-inflammatory, antibacterial, and wound-healing potential. However, critical evaluation reveals important methodological inconsistencies across studies that complicate the interpretation of results.

A 2013 study combined hypericum and neem oils and applied them topically to pediatric burns. This retrospective clinical review enrolled 9 pediatric patients with burn wounds (mean age 8.17 ± 3.35 years) (mean wound area 50.76 ± 48.32 cm^2^; range 4.63–132 cm^2^), with no randomized control group; the applied dose and frequency of application were not reported in the publication. The study found the combination reduces pain and effectively treats burnt soft tissue [9]. The authors described the therapeutic intent as primarily analgesic and antimicrobial; however, because neither the neem oil nor the hypericum oil component was analytically characterized, the separate contributions of each oil to the observed outcomes—and the specific phytochemical constituents responsible—were not experimentally resolved. Any mechanistic attribution to neem-derived constituents such as nimbidin or limonoids, or to hypericum-derived constituents, would be inferential rather than data-supported. While these findings provide early-phase clinical support, this study relied on neem oil—a lipid-rich fraction predominantly containing fatty acids and limonoids—rather than the aqueous or ethanolic leaf extracts used in most preclinical models. The absence of a randomized design and the small sample size limit its generalizability, yet it remains the only clinically oriented evidence for neem in burn wound management.

**Figure 7:**
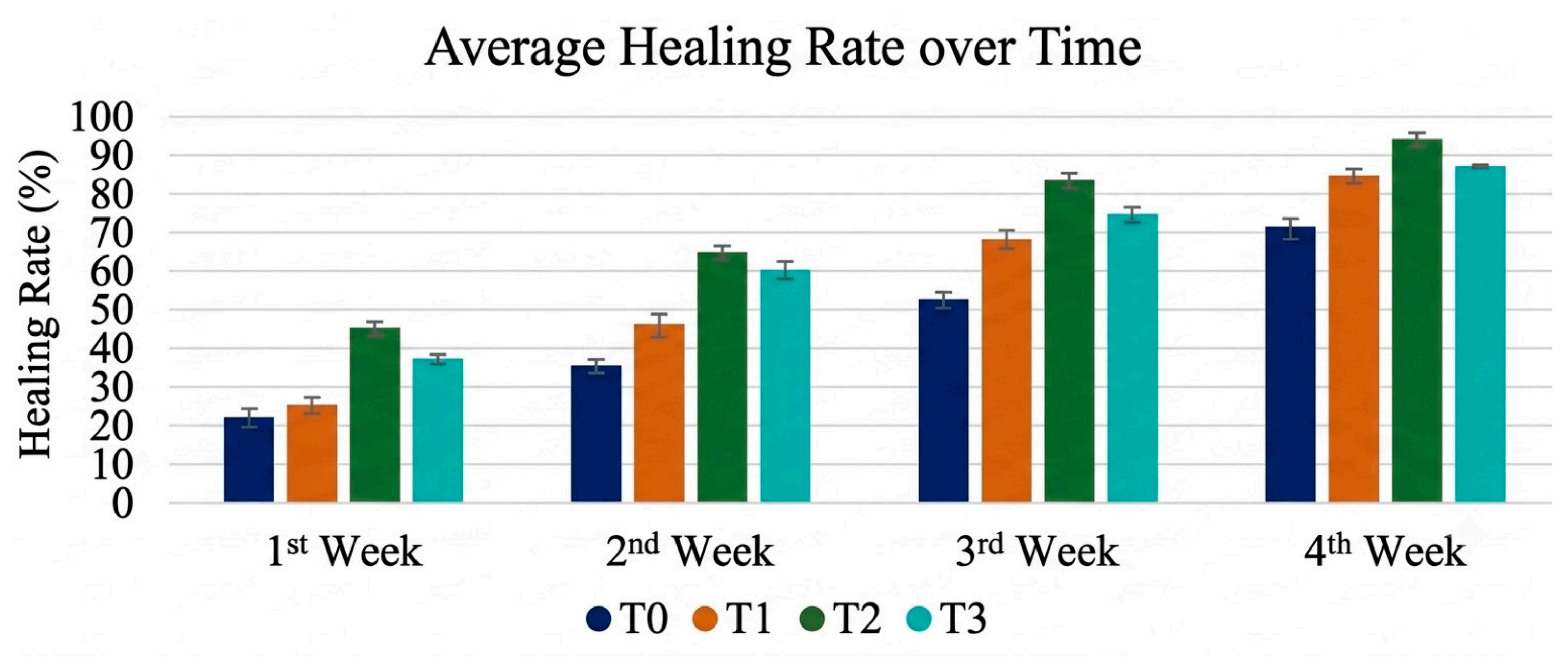
Comparative wound contraction and healing dynamics of contact thermal burn wounds in rabbits across experimental treatment groups. Healing rate (%) of hot-iron-induced burn wounds over a 4-week period in New Zealand White rabbits subjected to: T0 (untreated control), T1 (neem leaf extract alone), T2 (neem leaf extract combined with ZnO nanoparticles), and T3 (commercial Bactrocin^®^ ointment). Adapted from Rashid Anjum et al. [59] licensed under CC BY 4.0.

As an advanced treatment, a study in rabbits combining neem extract with zinc oxide nanoparticles showed that it significantly accelerated healing rates and reduced healing times compared to neem alone or commercial drugs such as Bactrocin^®^ (Figure 7) [59]. This study used 16 male New Zealand White rabbits (~2.5 months old; *n* = 4/group), with burn wounds induced by hot iron contact (wound depth not explicitly characterized in the publication). Neem leaf extract (4 g) combined with ZnO nanoparticles (1 g) was applied topically twice daily for 28 days, with wound healing assessed at weekly intervals (days 0, 7, 14, 21, and 28). The enhanced performance of the nanoparticle formulation likely reflects improved topical delivery and the independent antimicrobial contribution of zinc oxide, though the specific biological interactions remain to be delineated in larger animal models.

Similarly, formulations combining *Azadirachta indica* (AI) with other plant-derived substances, such as St. John’s wort oil or *Morus alba* (MA) extract, have shown enhanced granulation tissue formation and pain relief in burn wounds in rats, with the most significant results obtained with the MA + AI combination cream (93.03%) on day 11 [60]. This study employed 70 Wistar albino rats (175 ± 25 g, either sex; *n* = 10/group) using a scald model in which a shaved 20 mm^2^ dorsal area was submerged in boiling water at 90 °C for 15 s, which the authors classified as a deep second-degree to full-thickness burn based on exposure parameters. However, histological confirmation of full-thickness dermal necrosis was not established in the study protocol, so the burn depth cannot be reclassified confidently from the reported exposure parameters alone. Animals were treated for 21 days with either 20% *w*/*v* AI aqueous leaf extract cream, 20% *w*/*w* MA aqueous leaf extract cream, or a 10% + 10% MA + AI combination cream; application frequency was not explicitly reported, and wound contraction was traced planimetrically every 2 days. While enhanced healing was observed with the combination formulation, whether this reflects true phytochemical synergy or simple additive activity requires formal pharmacodynamic testing.

In contrast, the hypericum–neem oil combination employed in the pediatric clinical study [9] was associated with analgesia and antimicrobial protection, reflecting a different therapeutic intent; however, as noted above, the individual phytochemical contributions of neem oil versus hypericum oil were not resolved in that study either. These mechanistic and compositional differences across studies make direct efficacy comparisons unreliable.

A key source of discrepancy across burn wound studies lies in the heterogeneity of plant material and preparations used. Neem oil (used in the clinical pediatric study) differs substantially in its phytochemical profile from ethanolic or aqueous leaf extracts (used in most rodent studies) and nanoparticle-encapsulated preparations (used in the rabbit model). Doses and treatment durations also varied substantially across studies: the rabbit model used 4 g neem leaf extract combined with 1 g ZnO nanoparticles applied twice daily for 28 days [59]; the rat model used a 20% *w*/*v* AI leaf extract cream applied for 21 days, with wound contraction measured every 2 days [60]; and dose and application frequency were not reported in the clinical study [9]. This variation in the active compound profile—along with differences in animal species (rabbits vs. rats), burn induction methods (thermal contact vs. scald), wound depth characterization, and outcome endpoints (healing rate, wound closure percentage, histological scoring)—renders cross-study comparison difficult and explains much of the variability in reported efficacy values. To date, direct comparisons between neem oil and neem leaf extract in burn wound healing have not been identified among the studies reviewed, representing a notable and addressable gap in the literature.

Additional knowledge gaps further limit the strength of existing evidence. Because the reported scald and contact models lacked rigorous histological verification of complete dermal and adnexal destruction, definitive evidence evaluating neem specifically in histologically confirmed third-degree (full-thickness) or fourth-degree burns remains absent from the literature; the existing preclinical evidence is therefore confined to superficial and deep partial-thickness injuries. The effect of neem-based formulations on scar formation, collagen remodeling, and long-term tissue architecture has not been assessed. Furthermore, burn wounds are disproportionately colonized by clinically resistant organisms such as *Pseudomonas aeruginosa* and methicillin-resistant *Staphylococcus aureus* (MRSA); however, the studies identified in this review provide limited evidence regarding the antimicrobial efficacy of neem against these pathogens within a burn wound context. Finally, the safety of topical neem application on compromised burn skin barriers—particularly the risk of systemic absorption, sensitization, or allergic contact dermatitis—has not been adequately characterized in the reviewed studies. These gaps collectively indicate that while neem holds promise as an adjunctive agent in burn wound management, the evidence base is insufficient to support clinical recommendations without further targeted investigation (Table S1).

#### 4.5.3. Infected Wounds

An infected wound occurs when pathogens invade the skin or underlying tissue, causing inflammation, tissue damage, and delayed healing. Symptoms include redness, swelling, pain, pus formation, and fever [61]. The wound-healing properties of neem make it an interesting subject of study for infected wounds, given its antimicrobial and medicinal properties. However, a critical distinction must be drawn between basic in vitro antibacterial screening (e.g., minimum inhibitory concentration (MIC) assays or agar disk diffusion) and true in vivo infected wound models evaluating active tissue repair under physiological bioburden.

**Figure 8:**
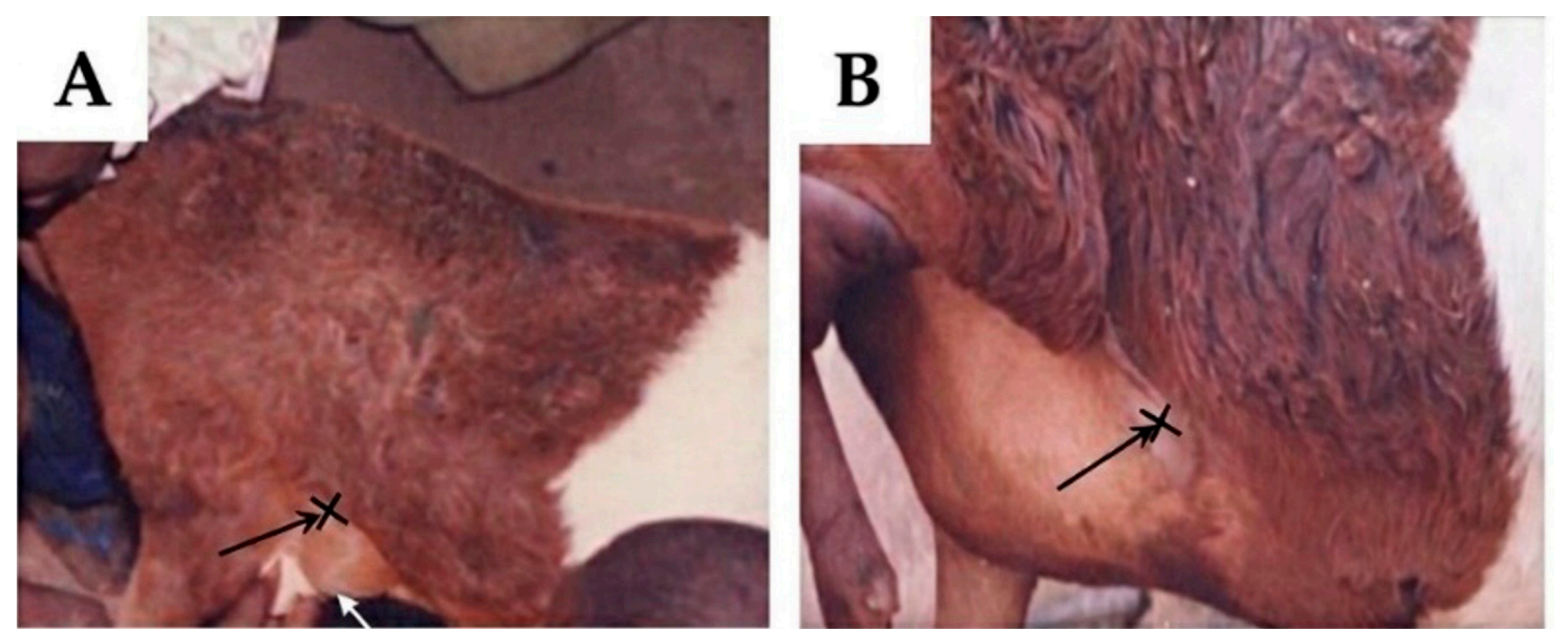
Therapeutic resolution of an infected surgical wound in a ram treated with topical crude neem seed oil. (**A**). Swelling in the ram’s wound indicates inflammation; the arrow points to the circumscribed swelling. (**B**). Wound closure completed within 7 days; the arrow points to the scar formed after the wound healed completely. Reproduced from Bwala et al. [62], licensed under CC BY 4.0.

In a veterinary clinical observation, Bwala et al. [62] reported that the topical application of crude neem oil on an infected surgical wound in a one-year-old Uda breed ram resulted in the complete re-epithelialization and closure of a 7 cm lesion within seven days (Figure 8A,B). The formulation was 100% locally processed crude oil derived from the neem seed kernel, administered as a single topical application on the day of surgery. Although wound closure was reported, this report represents a single uncontrolled veterinary case in one animal, which inherently limits its generalizability to human wound care. Furthermore, the use of crude seed kernel oil—rather than aqueous or ethanolic leaf extracts that dominate the wider neem wound healing literature—introduces substantial chemical heterogeneity, as neem seed oil is dominated by hydrophobic lipid matrices and limonoids rather than the hydrophilic polyphenolic constituents characteristic of leaf preparations. The absence of a non-treated control group, standardized bacterial inoculation protocol, or serial quantitative microbiological titrations makes it difficult to isolate neem’s antimicrobial efficacy from the host’s innate physiological healing capacity.

A recent study by Villani and colleagues developed neem-Hypericum-bacterial cellulose paste and evaluated its performance in both in vitro and in vivo models [63]. The in vivo component of this study, however, employed *Galleria mellonella* (wax moth) larvae—an invertebrate model—rather than a mammalian system. While *G. mellonella* provides a high-throughput, ethically viable platform for early antimicrobial screening, it lacks mammalian skin architecture, complex vascularization, and adaptive immune responses, precluding direct translational extrapolation to human cutaneous repair. In this model, a standardised thermal/mechanical lesion (~2 mm^2^) was created on the larval dorsum and inoculated with *Pseudomonas aeruginosa* and *Staphylococcus aureus*; application of the biocomposite paste (~100 mg per wound site) was one-hour post-infection significantly enhanced larval survival over a 4-day monitoring window. Furthermore, the neem oil utilized in the formulation lacked analytical characterization, with no quantitative profiling of marker limonoids or fatty acid standardization reported.

Arena et al. [42] conducted a randomized, single-blinded, parallel-group phase III trial of a hypericum–neem oil dressing, Primary Wound Dressing [ONE] (1PWD), versus silver-based dressings in dehisced surgical wounds with critical colonization or infection. Of 99 enrolled patients, 49 in the experimental group and 48 in the control group completed follow-up. Assessments were conducted at baseline and on days 7, 14, 21, and 28 using the Bates–Jensen Wound Assessment Tool (BWAT) and a numeric rating scale (NRS) for pain. The published abstract reported no statistically significant between-group difference in overall BWAT scores (*p* = 0.81) or the seven inflammatory/infective BWAT items (*p* = 0.35). Pain reduction favored the hypericum–neem dressing (*p* < 0.0001). These findings support a pain-related benefit of the co-formulation under the study conditions; non-significant BWAT differences do not establish equivalence or superior infection control. The trial does not isolate neem’s contribution, establish phytochemical synergy, or demonstrate microbiological eradication.

Despite these clinical advancements with co-formulations, a critical gap across all reviewed literature is the lack of pathogen-specific and biofilm-focused evaluations for pure neem treatments. Infected chronic wounds are frequently colonized by polymicrobial communities and mature biofilms comprising resistant Gram-positive (*S. aureus*, MRSA) and Gram-negative (*P. aeruginosa*, *E. coli*) pathogens. The studies reviewed do not establish how standardized neem preparations compare with established antimicrobial wound treatments in mature biofilm models. Such evaluations should include quantification of extracellular polymeric substance (EPS) matrices and viable bacterial burden.

Human randomized evidence discussed in this section concerns a hypericum–neem co-formulation [42]. It does not establish the efficacy of neem monotherapy in a microbiologically defined infected wound population. Further trials should distinguish clinical inflammation from microbiological infection, quantify bacterial burden and biofilms, and compare chemically characterized preparations with appropriate active dressings (Table S1).

#### 4.5.4. Incision and Excision Wounds

Incision and excision wounds are two common skin injuries that differ in their causes and characteristics. Incision wounds are cuts caused by sharp objects such as knives, scalpels, or broken glass. These wounds can vary in depth, from superficial cuts that affect only the outer skin to deeper injuries that involve muscles, tendons, or blood vessels [64]. In contrast, excision wounds involve removing tissue to eliminate diseased or damaged areas. These types of wounds require careful treatment to prevent infection and ensure proper healing [65].

**Figure 9:**
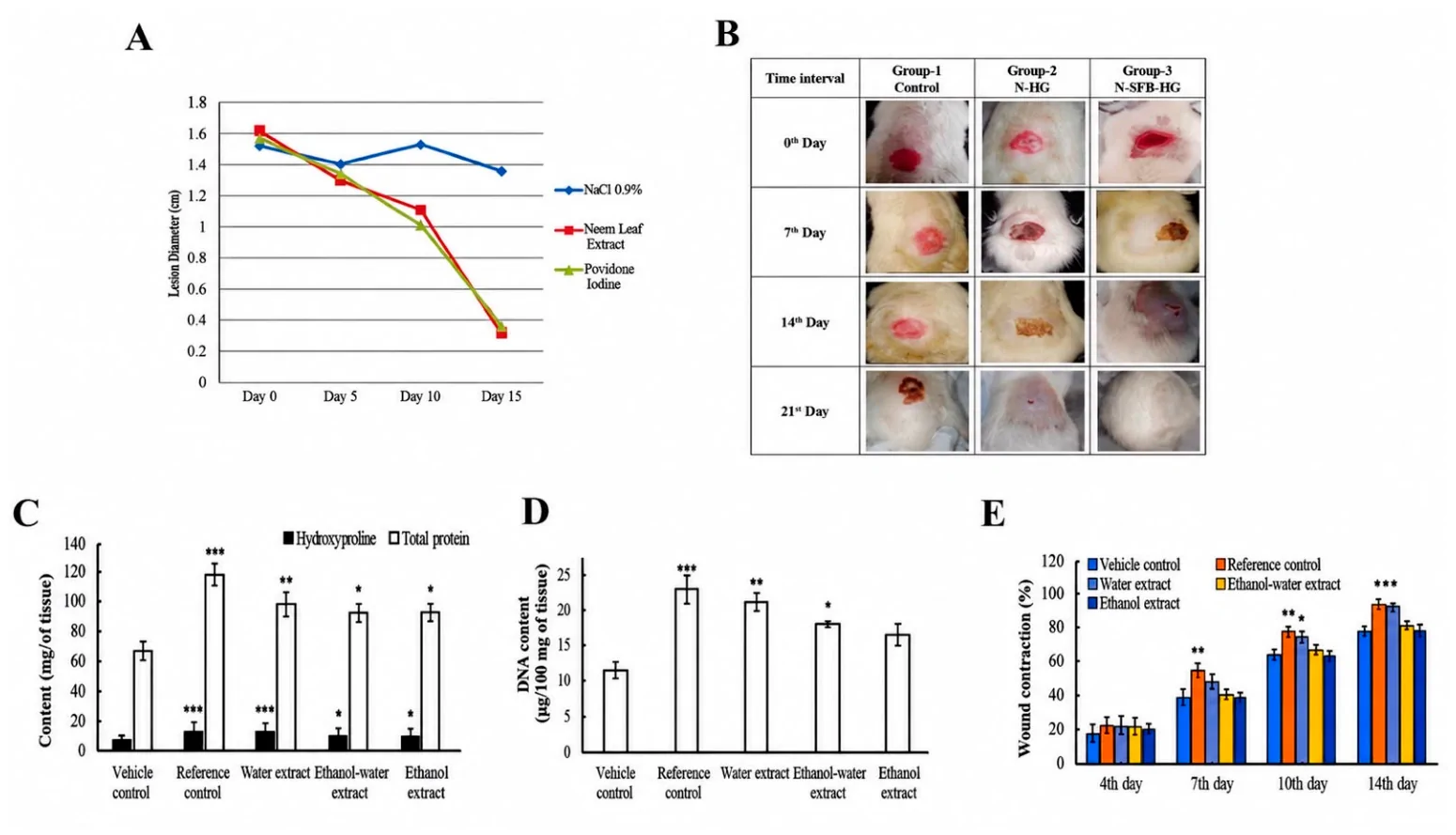
In vivo wound contraction kinetics, macroscopic healing progression, and biochemical markers of neem formulations in Incision and excision wound models. (**A**). The diameter length after treatment for over 15 days. Reproduced from Chundran et al. [43], licensed under CC BY-NC-SA 4.0. (**B**). Macroscopic wound closure progression in rats treated with neem hydrogel (N-HG) over 21 days. Reproduced without modification from Nasrine et al. [68], licensed under CC BY-NC-ND 4.0. Granulation tissue parameters in mice after 14-day treatment with stem bark extracts: (**C**). hydroxyproline and total protein levels, (**D**). DNA content, and (**E**). percentage wound contraction (* *p* < 0.05, ** *p* < 0.01, *** *p* < 0.001 vs. vehicle). Data represent mean ± SD. Adapted from Maan et al. [73], licensed under CC BY-NC-SA 4.0.

Different parts of the neem tree have been studied in incision and excision models for their wound-healing properties. While the overall body of evidence supports neem’s therapeutic potential across its various plant parts, a closer examination reveals notable inconsistencies that are attributable to differences in plant part used, extraction solvent, animal species, and wound model, each of which warrants critical discussion. A study of neem leaves has shown the remarkable efficacy of the leaf extract in excising wound models in rats, attributed to its anti-inflammatory, antimicrobial, and collagen-promoting properties. Specifically, the study used Wistar rats (approximately 200–250 g) with a 1.5 cm diameter full-thickness excision wound, applying a leaf extract obtained by maceration in 96% ethanol over a 15-day treatment period, with wound assessments performed on days 0, 5, 10, and 15. The extract reduced wound diameter relative to saline, with a healing pattern resembling that of the povidone-iodine group (Figure 9A) [43]. Attribution to nimbidin or sodium nimbidate remains inferential because these constituents were not quantified in the tested preparation.

Similarly, ethanolic neem leaf extract of 5% *w*/*w* (applied alone as an ointment) produced faster healing in both excision and incision wounds in rats, showing faster wound contraction than controls (simple ointment base) and standard (framycetin sulfate cream 1% *w*/*w*) [66]. The study used Wistar albino rats (180–250 g), with excision wounds treated once daily until complete epithelialization and incision wound tensile strength assessed on post-wounding day 10. Notably, this study employed a commercially sourced ethanolic extract obtained from the Himalaya Drug Company—the only commercial preparation among the studies reviewed in this section—which represents a closer approximation to a standardized product than laboratory-prepared extracts.

Polyherbal formulations and advanced delivery systems have also been investigated using excision wound models. Previous research evaluated a polyherbal ointment containing neem, *Salvadora persica*, and *Calendula officinalis* (10% *w*/*w* ointment applied once daily) on full-thickness excision wounds (approximately 150 mm^2^) in eighteen male Wistar rats, achieving complete wound closure within 19 days following treatment with the combination [67]. Furthermore, advanced delivery scaffolds such as neem-silk fibroin hydrogels (N-SFB-HG) and neem hydrogels (N-HG) demonstrated accelerated wound contraction and closure by day 21 in a 2 cm diameter full-thickness excision wound model in male Wistar albino rats [68]. The three-dimensional fibroin matrix functioned as a supportive physical delivery platform, maintaining a moist microenvironment and facilitating cellular migration within an uninfected wound bed, resulting in marked macroscopic re-epithelialization and accelerated scab detachment (Figure 9B) [68]. Separately, the formulation’s antimicrobial efficacy was evaluated through auxiliary in vitro assays, demonstrating notable inhibition against *Escherichia coli* and *Staphylococcus aureus* [68]. However, this antibacterial activity was determined exclusively in culture media and was not challenged against an established in vivo wound infection. Consequently, while these in vitro findings confirm the bioactivity of the released phytochemicals, the accelerated wound closure observed in the animal model represents biomaterial-guided structural tissue repair rather than infection resolution.

Extending polymeric biomaterial strategies to address acute surgical hemorrhage alongside tissue regeneration, Rahman and Mondal [69] engineered a hydrophobic biocomposite film incorporating *Azadirachta indica* seed oil within a cross-linked polyvinyl alcohol (PVA)/chitosan polymeric matrix. The resulting biocomposite film displayed optimal surface hydrophobicity (water contact angle: 108.4°), balanced water vapor transmission (2150 g/m^2^·day), and rapid hemostatic activity, with a reported in vitro blood clotting index (BCI) of 91.44% within 5 min compared to plain gauze controls. In an in vivo full-thickness excision rat model, the neem-oil-loaded composite dressing significantly accelerated tissue repair (complete closure by day 16, *p* < 0.001), while histological examination revealed advanced structural maturation characterized by continuous stratified re-epithelialization, high fibroblast cellularity, well-organized collagen bundle deposition, and the de novo regeneration of dermal appendages including hair follicles.

In direct contrast, a 2020 study found that topical application of aqueous neem leaf extract and neem ointment reduced inflammation but was ineffective at accelerating wound healing in sheep [70]. The model used adult Santa Inês sheep with a large 7 × 7 cm full-thickness excision wound on the chest wall, evaluated over 28 days at days 7, 14, 21, and 28; notably, the exact concentrations of the neem preparations applied were not reported by the authors, a methodological limitation that reduces reproducibility and complicates cross-study dose comparisons. This represents a significant and unresolved discrepancy when compared to the consistently positive findings in rat models, and several factors may account for this divergence. First, aqueous extraction is considered—based on general solubility principles and the broader phytochemical literature on neem—to be less efficient at solubilizing the lipophilic terpenoids and flavonoids that have been associated with wound-healing activity in neem. Second, species-specific differences in wound healing physiology between rats and sheep—including differences in skin anatomy, immune response kinetics, and inflammatory resolution—may modulate the responsiveness to neem’s bioactive constituents. Third, the rodent model relies heavily on rapid wound contraction mediated by the panniculus carnosus, a process whose contribution differs substantially among species, suggesting that efficacy in rodents may not reliably predict outcomes in sheep or humans. This discrepancy has not been resolved by the comparative evidence reviewed here and represents one of the most important unresolved questions in this area.

In 2018, a study compared topical and oral administration of the leaf extract in rats with excision wounds. The study used albino rats (90–125 g) with a 1 cm diameter excision wound; the oral group received 100 g of ground neem leaves incorporated into 1000 g of standard feed, and wound closure was monitored every alternate day over up to 14 days (days 0, 2, 4, 6, 8, 10, 12, and 14). The group treated topically with neem extract showed a 92% contraction rate (*p* < 0.05) and a shorter wound closure time compared to the orally fed group [71]. Topical application provides direct exposure to the damaged tissue interface, avoiding first-pass hepatic metabolism and systemic degradation that could potentially reduce oral bioactivity. However, because specific pharmacokinetic parameters and tissue bioavailability were not directly quantified, these pharmacological factors remain plausible working hypotheses. This distinction should inform future formulation strategies and discourages the assumption that systemic neem supplementation can substitute for topical delivery in wound care.

Neem-based ZnO and Ag-ZnO NPs loaded into chitosan hydrogel were applied to wounded male albino rats (~250 g; 2 cm diameter full-thickness excision wounds; wound area monitored on days 0, 4, 8, and 14 over a 14-day treatment period) and showed promising results, with reported improvements in wound healing. The chitosan-based dressing played an important role in providing a moist environment through moisture retention and exudate management, which stimulates wound epithelialization [72]. While the results are encouraging, the individual contribution of neem’s phytochemicals to the observed outcomes is difficult to isolate in this formulation, given the well-established independent wound-healing and antimicrobial activities of both zinc oxide nanoparticles and chitosan. Future studies should include neem-free chitosan-ZnO control arms to delineate the specific added value of neem in such composite systems.

A 2017 study used different neem bark extracts (water, ethanol, ethanol–water) applied topically as an ointment (15% *w*/*w*) in excision and incision wound models. The study used Swiss albino mice (23 ± 3 g); excision wounds (177 mm^2^) were treated with 30 mg of ointment once daily for 14 days and assessed on days 0, 4, 7, 10, and 14, while incision wound breaking strength was measured on day 10. Water extracts of the stem bark improved wound contraction, hydroxyproline content, and DNA content in mouse models (Figure 9C–E) [73]. This finding is notable when contrasted with the aqueous leaf extract study in sheep [70], which found aqueous preparations ineffective. A distinction between the two has been proposed on the basis of the general phytochemical literature on *A. indica*: neem bark is reported to be particularly rich in water-soluble tannins and polysaccharides associated with astringent and pro-collagen synthesis properties, while the aqueous leaf fraction is considered relatively depleted in the lipophilic terpenoids most commonly associated with wound-healing activity.

Similarly, a study compared the effects of neem stem bark extract with povidone-iodine ointment. The study employed albino/Wistar rats (170–210 g) with a 5 mm^2^ full-thickness excision wound, applying a 10% *w*/*w* ethanolic or 5% *w*/*w* aqueous bark ointment once daily for up to 15 days, with wound assessments on days 1, 3, 6, 9, 12, and 15. The study found that neem extract has a better and faster wound-healing effect than the standard drug, povidone-iodine ointment, in the excision wound model [74]. While this comparison is favorable, it should be interpreted with caution: povidone-iodine is primarily an antiseptic agent rather than a regenerative wound-healing treatment, and benchmarking against it does not reflect neem’s efficacy relative to modern advanced wound dressings or regenerative standards of care.

A recent study by Ajayi and Omolere concluded that neem seed (5–10% *v*/*v*) hexane and methanolic extracts significantly accelerated wound contraction in a rat excision model through their bioactive compounds. The study used female Wistar albino rats (110–245 g); seed extracts were formulated as 5% and 10% *v*/*v* ointments in soft yellow paraffin and assessed over 21 days, with wound measurements on days 1, 4, 8, 12, 16, and 20. The 5% neem methanol extract ointment showed the fastest wound healing compared to the control ointment [75]. The seed’s phytochemical profile—dominated, according to the general neem phytochemical literature, by azadirachtin, fatty acids such as oleic and stearic acid, and limonoids—differs substantially from that of leaves or bark. The superior performance of the methanolic over hexane extract suggests that polar bioactive compounds in neem seeds contribute more substantially to wound healing than the lipophilic hexane-extractable fraction; however, because neither extract was subjected to analytical characterization, the specific polar compounds responsible for the observed efficacy remain unidentified, and any attribution to azadirachtin, flavonoids, or other constituent classes would be speculative rather than experimentally grounded. Furthermore, the superior performance of 5% over 10% mirrors the non-linear dose–response pattern observed in other neem wound studies across different wound types, consistent with a possible cytotoxic or pro-inflammatory threshold effect at higher concentrations—a pattern that deserves systematic investigation.

Sahni and Dubey [52] reported an uncontrolled study of 20 patients with mixed wounds, including five incised wounds and one burn. Patients received oral neem powder containing leaves, stem and root bark, flowers, and fruits (3 g twice daily for 30 days), together with topical neem leaf decoction on alternate days. Wounds were traced manually, and severity scores were compared before and after treatment. The reported wound-size score decreased from 1.70 to 0.15, corresponding to 91.17% improvement; this is a score-based result and should not be interpreted as a directly measured 91.17% reduction in wound area. Pain and other symptom scores also improved (reported *p* < 0.001). Ten patients were classified as markedly improved and ten as moderately improved; none met the study’s definition of cure. The absence of a control group, mixed etiologies, and combined oral and topical treatment limit causal interpretation and prevent estimation of a separate effect in incised wounds.

Across the full body of evidence for incision and excision wounds, several critical knowledge gaps emerge. Taken together, the reviewed studies demonstrate substantial methodological heterogeneity: animal species ranged from Wistar and albino rats (90–250 g) and Swiss albino mice (23 ± 3 g) to Santa Inês sheep (~30 kg); wound areas spanned from 5 mm^2^ to 49 cm^2^ (7 × 7 cm); treatment durations ranged from 10 to 28 days; and plant parts (leaves, stem bark, seeds) were extracted with different solvents (water, 96% ethanol, hexane, methanol, or combined) and applied at concentrations ranging from 5% to 15% *w*/*w*. No two studies employed identical experimental conditions, rendering direct inter-study comparison methodologically unsound and substantially limiting the strength of any cross-preparation conclusions. First, the studies identified in this review did not directly and systematically compare leaves, bark, and seeds under identical experimental conditions and wound models, making it impossible to determine which plant part offers the greatest therapeutic efficacy. Second, the conflicting results between aqueous and ethanolic leaf extracts across species have not been directly investigated; the relative roles of extraction solvent, phytochemical composition, and animal species in this discrepancy remain unresolved. Third, the mixed clinical cohort [52] did not report outcomes separately for incised wounds. The randomized study of infected or critically colonized surgical dehiscence [42], discussed in Section 4.5.3, addresses a different clinical setting and a co-formulated product. Neither establishes the efficacy of neem alone in uncomplicated surgical incisions or excisions. Fourth, outcome metrics—including hydroxyproline content, wound contraction rate, and epithelialization time—are reported inconsistently across studies, preventing meaningful meta-analytic comparison. Finally, long-term outcomes such as scar quality, tensile strength of healed tissue, and recurrence of infection remain inadequately characterized in the studies discussed here, all of which are clinically important parameters in surgical wound management. Addressing these gaps through standardized, comparative, and clinically oriented research is essential to establishing neem’s role in evidence-based incision and excision wound care (Table S1).

### 4.6. Analytical Standardization as a Principal Translational Barrier

A critical evaluation across preclinical and clinical wound-healing investigations reveals that the lack of rigorous phytochemical standardization represents one of the principal impediments to reproducibility and translational advancement. Across the reviewed literature, biological activities—ranging from accelerated re-epithelialization and angiogenesis to anti-inflammatory and antimicrobial effects—have been predominantly attributed to crude, unstandardized preparations [5,6,9,63]. In many of the reviewed extract studies, specific active constituents (such as nimbidin, nimbin, azadirachtin, or quercetin) were inferred from historical phytochemical literature rather than analytically quantified in the experimental batches tested [43,60,67,73].

This widespread methodological omission introduces several confounding variables that obscure true structure–activity and dose–response relationships:

Plant Part and Solvent Divergence: Neem oil, leaf extracts, bark extracts, and gum-based scaffolds are chemically distinct preparations. Their composition depends on botanical material and processing, and constituent profiles reported for one preparation cannot be assumed for another. The studies include ethanolic leaf extracts [43,54], aqueous leaf preparations [5,52], seed oil preparations [6,9,42], and methanolic or hexane seed extracts [75]. The oral multi-part powder and topical leaf decoction used by Sahni and Dubey further complicate attribution to a particular plant part or route [52]. Reproducibility requires reporting the plant-to-solvent ratio, extraction temperature and duration, yield, and batch-specific chemical characterization.

Batch-to-Batch Heterogeneity: Phytochemical yields are inherently susceptible to seasonal variations, geographical origins, harvest maturity, and drying protocols. When plant materials are sourced locally without quality specifications [59,63], reported biological responses cannot be isolated from natural agro-climatic variability, preventing true experimental reproducibility across independent laboratories.

Non-linear Dose Dynamics: Multiple preclinical models demonstrated non-linear or paradoxical dose–response profiles. For instance, in diabetic excision models, a 10% foliar extract in virgin coconut oil outperformed a 15% formulation in reducing IL-6 and accelerating wound closure [54]. Similarly, a 5% methanolic seed extract accelerated wound contraction faster than a 10% concentration [75]. Chemical profiling alone cannot establish the mechanism of a non-linear response. Formulation studies, release measurements, and dose-dependent cytocompatibility testing are also needed to distinguish vehicle effects from pharmacological or toxicological explanations.

Ambiguous Topical Dose Metrics and Matrix Interactions: Dosing protocols across the identified literature are almost universally reported as crude weight-to-volume (*w*/*v*) or weight-to-weight (*w*/*w*) percentages, or vague volumetric rinses. None of the studies quantified topical delivery normalized to the wound surface area (e.g., mg or µg of quantified marker constituent per cm^2^), obscuring the actual pharmacological threshold delivered to the healing tissue bed. Furthermore, while modern composite delivery systems—such as electrospun polysaccharide nanofibers [57] and polymeric biocomposite films or hydrogels [68,69]—successfully manage fluid kinetics, wound exudate, and rapid hemostasis, reported release measurements do not necessarily characterize the delivery of neem-derived constituents. For example, curcumin release from a neem-gum scaffold [57] should not be equated with quantified release of neem limonoids.

To improve reproducibility and facilitate future translational research, subsequent wound-healing studies should prioritize the chemical characterization and standardization of neem preparations. Rather than requiring immediate development as botanical drug substances, early research should establish consistent extraction procedures, phytochemical profiles, and biologically relevant dose ranges. Potential analytical approaches include chromatographic and spectrometric profiling, such as High-Performance Liquid Chromatography with Diode-Array Detection (HPLC-DAD), liquid chromatography–tandem mass spectrometry (LC-MS/MS), or Gas Chromatography–Mass Spectrometry (GC-MS), depending on the extract type and study objectives. Where applicable, marker compounds such as azadirachtin, nimbin, or nimbidin may be used to support batch characterization. However, the selection of markers should be justified according to the plant part, extraction method, and chemical composition of the preparation. Good Manufacturing Practice (GMP) compliance and regulatory requirements may become relevant during later stages of product development. At the preclinical research stage, the immediate priority is to report sufficient methodological and chemical information to support reproducibility, comparison between studies, and appropriate interpretation of biological findings. The preclinical and clinical evidence for neem formulations across wound types is summarized in Table 3.

## 5. Limitations and Challenges

The wound-healing properties of neem, supported by various studies, still face limitations and challenges that prevent their widespread clinical application.

Methodological limitations are common across the reviewed studies. Most investigations rely on small sample sizes and preclinical animal models, making it difficult to translate the outcomes to clinical application. Furthermore, wound healing is frequently evaluated primarily through macroscopic wound closure kinetics; where histological assessments are conducted to confirm re-epithelialization, collagen bundle deposition, and dermal appendage regeneration, they predominantly remain qualitative and descriptive rather than employing standardized semi-quantitative histopathological scoring systems (e.g., validated scales for inflammatory cell infiltration, fibroblastic cellularity, or stereological microvessel density) [5,6,9,63,69]. In addition, many studies lack standardized objective quantitative outcome measures, such as digital wound planimetry, validated healing-rate calculations, and biochemical assessment of collagen synthesis and inflammatory biomarkers, reducing the robustness, reproducibility, and credibility of the reported findings.

Considerable heterogeneity in neem plant parts, extraction methods, formulations, animal species, and wound models further limits comparisons among studies and the generalizability of results. Moreover, evidence remains limited regarding the comparative efficacy of different neem plant parts and extraction methods under standardized experimental conditions, making it difficult to identify the most preferred therapeutic preparation. Non-linear dose–response relationships have also been reported, with intermediate concentrations often outperforming higher doses, suggesting potential concentration-dependent cytotoxic or pro-inflammatory effects that require further investigation.

Comparisons with active wound-care products remain uneven. Arena et al. compared a hypericum–neem dressing with silver-based dressings in a randomized trial [42]; it would therefore be inaccurate to state that clinical comparisons with active dressings are entirely absent. However, this trial cannot establish neem-specific efficacy, and the uncontrolled mixed-wound study by Sahni and Dubey provides no comparative estimate [52]. Neither study establishes pharmacological synergy. Additional limitations include short follow-up, heterogeneous wound etiologies, and insufficient evidence for resistant pathogens, mature biofilms, scar quality, and long-term tissue strength.

Safety remains formulation- and exposure-dependent. Manca et al. evaluated neem oil dispersions and phospholipid vesicles in cultured keratinocytes and fibroblasts at 1, 10, and 20 µg/mL using cell-viability assays and a scratch model [76]. Viability results should not be reported as a quantified apoptosis rate or attributed to a specific membrane-disrupting mechanism without direct measurements. These experiments support further formulation research but do not establish safety on human open wounds. Oral and systemic toxicology reports also cannot establish cutaneous safety across a damaged skin barrier [77,78]. Short-term clinical observations with the regimens in Refs. [42,52] do not resolve sensitization, percutaneous absorption, repeated-exposure toxicity, or safety in vulnerable populations. Dedicated testing of the final topical preparation and clinically relevant exposure conditions remains necessary.

Overall, while preclinical findings are encouraging, the current evidence remains insufficient to support definitive clinical application, highlighting the need for rigorous, standardized human studies before neem can be considered an evidence-based wound therapy.

## 6. Future Directions

Current research on neem and wound healing is progressively shifting toward the development of advanced biomaterial delivery platforms. This includes the fabrication of functionalized scaffolds engineered to preserve phytochemical stability and achieve sustained, spatiotemporally controlled release of neem bioactives directly into the wound microenvironment. For example, stimuli-responsive hydrogels that adapt dynamically to localized physiological cues—such as pH fluctuations, elevated protease activity, or temperature shifts—exhibit substantial clinical potential. Additionally, macroporous cryogels offer distinct structural advantages over conventional hydrogels due to their interconnected porous architecture, high exudate absorption capacity, and the possibility of retaining useful properties after autoclaving in selected polymer systems [79]. Beyond hydrogels, the integration of neem bioactives into electrospun polysaccharide nanofibers and hydrophobic biocomposite films represents a promising horizon for combining rapid surgical hemostasis with accelerated tissue regeneration.

Despite these advances, several important challenges must be addressed before neem-based therapies can be translated into routine clinical practice. Rigorous phytochemical standardization of extraction methodologies and final formulations is essential to reduce and control batch-to-batch variability and establish reproducible target profiles for critical marker constituents. Furthermore, comprehensive safety profiling remains imperative; current literature severely lacks robust evaluations of percutaneous penetration across compromised dermal barriers, localized sensitization and allergic contact dermatitis, long-term phototoxicity, and pharmacokinetic characterization in vulnerable clinical populations, such as pediatric and geriatric patients with extensive burn or ulcer surfaces.

Future investigations must also transition from qualitative macroscopic observations to standardized quantitative histological scoring alongside molecular profiling. Deepening the mechanistic evidence base requires elucidating neem’s precise modulation of canonical wound-healing signaling cascades, specifically the NF-κB/IκB signaling axis in persistent inflammation, the Nrf2/HO-1 pathway in oxidative stress abatement, and the TGF-β/Smad and VEGF/VEGFR2 axes governing ECM remodeling and functional angiogenesis.

Finally, bridging the translational divide fundamentally demands well-designed, prospective, double-blind randomized controlled clinical trials with blinded outcome assessment where feasible benchmarking standardized neem formulations against contemporary advanced dressings and bioengineered grafts.

Parallel optimization of scalable manufacturing processes under Good Manufacturing Practice (GMP) regulations, coupled with stringent regulatory harmonization, will be pivotal in translating neem from an empirical ethnomedicinal remedy into an evidence-based, cost-effective, and globally accessible therapeutic modality for complex wound management.

## 7. Conclusions

Chronic wounds that fail to progress through physiological repair cascades represent a persistent clinical burden, frequently predisposing patients to systemic infection, extensive tissue necrosis, or limb loss. Preclinical data across diverse in vitro and in vivo animal models lend strong biological plausibility to the wound-healing activity of *Azadirachta indica* (neem), largely mediated by its multi-target modulation of persistent inflammation, oxidative stress, bacterial bioburden, and cellular proliferation. However, these preclinical observations must not be interpreted as definitive evidence of established clinical efficacy. The current body of literature remains predominantly preclinical, marked by substantial methodological heterogeneity, reliance on small-animal models, and an overreliance on qualitative descriptive histology rather than standardized, semi-quantitative histopathological scoring systems. Human findings include a randomized comparison of a hypericum–neem dressing with silver-based dressings [42] and an uncontrolled mixed-wound study using oral and topical neem [52]. These provide clinical observations, but do not establish the independent efficacy of topical neem across wound types.

Critically, a major translational bottleneck is the lack of chemical and batch-to-batch standardization among the crude extracts, fractions, and polyherbal formulations reported to date. Moving forward, modern drug-delivery platforms—such as stimuli-responsive hydrogels, macroporous cryogels with formulation-specific sterilization compatibility, and electrospun nanofibrous meshes—offer viable pathways to optimize the pharmacokinetic stability, bioavailability, and spatiotemporal release of neem-derived bioactive constituents. Nonetheless, meaningful clinical translation cannot proceed without two fundamental prerequisites: first, rigorous analytical standardization and chemical profiling of neem extracts to ensure reproducibility; and second, adequately powered, well-controlled randomized controlled trials benchmarking standardized formulations against current clinical standards of care. Ultimately, while preclinical findings support neem as a promising candidate for tissue repair, its definitive therapeutic utility and safety profile in human clinical practice remain to be substantiated through standardized, evidence-based clinical validation.

## Figures and Tables

**Table 1:** Summary of neem plant parts, their bioactive compounds, and functions.

Plant Part	Bioactive Compounds	Wound-Healing Relevant Activities
**Leaves**	Nimbin Nimbidin Nimbanene 6-desacetylnimbinene Nimbandiol Nimbolide Ascorbic acid N-hexacosanol Amino acid 7-desacetyl-7-benzoylazadiradione 7-desacetyl-7-benzoylgedunin 17-hydroxyazadiradione Nimbiol Quercetin, ß-sitosterol, Polyphenolic flavonoid [36]	Antimicrobial Antifungal Anti-inflammatory Immunomodulatory [38,39] Antioxidant [40]
**Seeds**	Azadirachtin, Gedunin, Nimbin, Nimbidin Nimbolides Salannin Tignic acid Meliantriol [36,41]	Antimicrobial Anti-inflammatory [42]
**Bark**	Triterpenoids Phenolic compounds Flavonoids Nimbidin [41]	Antioxidant Antimicrobial [38]
**Flowers**	N-Hentriacontane N-Nonacosane N-Pentacosane Methyl octadecanoate acid 2-Methoxy-5,40-dimethyl-benzenebutanal [41]	Antioxidant [38]
**Oil**	Azadirachtin Gedunin Salannin Nimbidin Meliantriol [41]	Antioxidant [38] Antimicrobial Anti-inflammatory [42]

Note: Bioactive compounds listed represent major phytoconstituents detected in the respective plant parts. The listed activities summarize reports for neem constituents or preparations; they do not establish wound-healing efficacy for every listed compound or plant part.

**Table 3:** Summary of preclinical and clinical evidence for neem formulations across wound etiologies.

Wound Category	Clinical and Preclinical Evidence	Comparators	Principal Reported Findings	Main Limitations
Diabetic wounds	DFU irrigation reports [5,8]: 200 enrolled and 160 analyzed in [5]. Mixed chronic-wound study: 20-patient diabetic subgroup [6]. Rodent studies [54,55,56,57].	Human: normal saline [5]; topical neem oil, oral turmeric, and their combination [6]. Animal comparators varied.	Improved wound-area and PUSH outcomes reported with neem irrigation [5]. Combination-arm healing signal in a small diabetic subgroup [6]. Animal studies reported contraction and tissue-repair outcomes.	Non-randomized clinical evidence; attrition and baseline imbalances; possible overlap between reports [5,8]; small subgroup; heterogeneous preparations.
Burn wounds	Uncontrolled pediatric series: *n* = 9, hypericum–neem oil [9]. One burn case in the mixed cohort [52], without separate results. Rabbit/rat studies [59,60].	No concurrent comparator in the pediatric series [9]. Animal studies included active topical comparators [59,60].	Clinical reports describe pain relief and healing with a co-formulation [9]. Preclinical combinations improved contraction in the tested models [59,60].	Small uncontrolled clinical series; no neem-specific effect; single burn case not independently interpretable; uncertain histological burn-depth classification.
Infected or critically colonized wounds	Human RCT of hypericum–neem dressing: 99 enrolled, 97 completed [42]. Veterinary case [62]; larval infection model and in vitro assays [63].	Human RCT: silver-based dressings [42]. Preclinical and veterinary comparisons differ in purpose and design.	RCT: no significant between-group BWAT difference; pain reduction favored the co-formulation [42]. Veterinary closure and larval survival were reported [62,63].	Co-formulation prevents neem-specific attribution; 28-day clinical follow-up; non-significance does not demonstrate equivalence or microbial eradication; limited mature-biofilm evidence.
Incision and excision wounds	Uncontrolled mixed cohort: *n* = 20, including five incised wounds [52]. Animal studies [43,66,67,68,69,70,71,72,73,74,75]. Surgical-dehiscence RCT [42] is summarized in the infected-wound row.	Mixed cohort: none [52]. Animal studies used vehicle or active topical comparators, including povidone-iodine and framycetin [43,66,67,68,69,70,71,72,73,74,75].	Mixed cohort: improved severity scores, without incision-specific outcomes [52]. Animal studies reported contraction, breaking strength, and tissue maturation; sheep findings were negative for accelerated healing [70].	Mixed etiologies and combined oral/topical exposure; no separate incision estimate; model and dose heterogeneity; limited long-term outcomes. The RCT [42] is not an independent additional cohort in this row.

## Data Availability

No new data were created or analyzed in this study. Data sharing is not applicable to this article.

## Supplementary Materials

The following supporting information can be downloaded at: https://www.mdpi.com/article/10.3390/ijms27219591/s1.

## Abbreviations

The following abbreviations are used in this manuscript:

1PWD	Primary Wound Dressing (ONE)
AI	*Azadirachta indica*
AI-AgNPs	*Azadirachta indica*-silver nanoparticles
AIG	*Azadirachta indica* gum
AIG-NF	*Azadirachta indica* gum nanofibers
ASC	Apoptosis-associated speck-like protein containing a CARD
ATP	Adenosine triphosphate
BCI	Blood clotting index
BWAT	Bates–Jensen Wound Assessment Tool
CI	Confidence interval
COX-2	Cyclooxygenase-2
CUR	Curcumin
DCF-DA	Dichlorofluorescein diacetate
DFUs	Diabetic foot ulcers
DNA	Deoxyribonucleic acid
DPPH	2,2-Diphenyl-1-picrylhydrazyl
ECM	Extracellular matrix
ELISA	Enzyme-linked immunosorbent assay
eNOS	Endothelial nitric oxide synthase
EPS	Extracellular polymeric substance
GC-MS	Gas Chromatography–Mass Spectrometry
GMP	Good Manufacturing Practice
H&E	Hematoxylin and eosin
HbA_1_c	Glycated hemoglobin
HO-1	Heme oxygenase-1
HPLC-DAD	High-Performance Liquid Chromatography with diode-array detection
IKK	IκB kinase
IL-1β	Interleukin-1β
IL-6	Interleukin-6
iNOS	inducible nitric oxide synthase
IP	Intraperitoneal
ITT	Intention-to-treat
LC-MS	Liquid Chromatography–Mass Spectrometry
LC-MS/MS	Liquid chromatography–tandem mass spectrometry
LOX	Lipoxygenase
LPS	Lipopolysaccharide
LD50	Median Lethal Dose (Lethal Dose, 50%)
MA	*Morus alba*
MAGS	Microscopic Angiogenesis Grading System
MIC	Minimum inhibitory concentration
MPO	Myeloperoxidase
MRSA	Methicillin-resistant *Staphylococcus aureus*
MyD88	Myeloid differentiation primary response 88
N-HG	Neem hydrogel
N-SFB-HG	Neem–silk fibroin hydrogel
NaCl	Sodium chloride
NF-κB	Nuclear factor kappa B
NIG	Nigericin
NLEHP	Neem leaf extract hydrogel patch
NLRP3	NOD-like receptor protein 3
NO	Nitric oxide
NPs	Nanoparticles
Nrf2	Nuclear factor erythroid 2-related factor 2
NRS	Numeric rating scale
PDGF	Platelet-derived growth factor
PGE_2_	Prostaglandin E_2_
PUSH	Pressure Ulcer Scale for Healing
PVA	Polyvinyl alcohol
PWAR	Percentage wound area reduction
ROS	Reactive oxygen species
SD	Standard deviation
Smad	Mothers against decapentaplegic homolog
SEM	Scanning Electron Microscopy
SSD	Silver Sulfadiazine
STZ	Streptozotocin
TGF-β	Transforming growth factor-β
TLR2	Toll-like receptor 2
TLRs	Toll-like receptors
TNFRs	Tumor necrosis factor receptors
TNF-α	Tumor necrosis factor-α
VCO	Virgin coconut oil
VEGF	Vascular endothelial growth factor
VEGFR2	Vascular endothelial growth factor receptor 2
VLUs	Venous leg ulcers
WHO	World Health Organization
XRD	X-ray Diffraction
ZnO	Zinc oxide
