# Supplementary material for: Azadirachta indica (Neem) in Wound Healing: A Review of Bioactive Compounds, Mechanisms, and Current Translational Evidence
Source: Int J Mol Sci. 2026 Oct 27;27(21):9591. doi: 10.3390/ijms27219591 (PMC13762628; doi:10.3390/ijms27219591)
Supplement: Supplementary file 1 [file ijms-27-09591-s001.zip › ijms-4427380-supplementary.pdf]

Azadirachta indica (Neem) in Wound Healing: A Review of Bioactive Compounds, Mechanisms, and Current Translational Evidence

Rafa Alomari [1], Manal El-Zohri [1,2] and Suzan Alharbi [1,*]

[1] Department of Biological Sciences, Faculty of Science, King Abdulaziz University, Jeddah 21488, Saudi Arabia; ralomari0064@stu.kau.edu.sa (R.A.); mnzohri@aun.edu.eg (M.E.-Z.)
[2] Department of Botany and Microbiology, Faculty of Science, Assiut University, Assiut 71516, Egypt
* Correspondence: saalharbi@kau.edu.sa

Supplementary Table S1. Methodological profiling, formulation parameters, translational outcomes, and risk-of-bias limitations of included studies.

Study, Etiology & Design	Model, Test System & Comparator	Plant Material, Extraction & Formulation	Dose / Regimen & Duration	Principal Quantitative & Histological Outcomes (Empirical)	Phytochemical Profiling & Standardization	Major Methodological Limitations Identified
**Jayalakshmi *et al.* [5] Diabetic Wounds Quasi-experimental clinical trial**	**Model:** Chronic DFUs (Wagner I-III). **Subjects:** Human patients (N=200 enrolled, n=160 completed). **Comparator:** Normal saline (0.9% NaCl)	**Material:** Fresh leaves. **Extraction:** Aqueous decoction. **Formulation:** Topical irrigation fluid via 19G needle	10 mL per ulcer bed, twice weekly for 4 weeks	Mean ulcer area reduced from 17.14 to 7.04 cm² (58.9% vs. 27.9% in saline; $p<0.001$); PUSH score improved. 82.3% achieved ≥50% PWAR	**Profiling:** None reported. **Standardized:** No (crude aqueous extract)	Non-randomized design; significant baseline disparities; normal saline comparator rather than active advanced dressings; 20% attrition without ITT analysis
**Singh *et al.* [6] Diabetic Wounds Prospective 3-arm clinical study**	**Model:** Chronic lower extremity ulcers. **Subjects:** Mixed chronic wounds (N=60 enrolled, DFU subset n=20). **Comparator:**	**Material:** Commercial seed oil. **Extraction:** Cold mechanical pressing. **Formulation:** Topical crude oil alone or	Topical dressing changed every 48h; Haridra oral 1g TID. Assessed at weeks 4, 8, 12	In DFU subset at 8 weeks: 75% achieved ≥50% closure vs. 40% (oil alone) and 0% (Haridra alone) ($p<0.05$); MAGS score	**Profiling:** None reported. **Standardized:** No (commercial crude oil)	Small DFU subgroup sample size; absence of ITT analysis for dropouts; lack of contemporary standard-of-

	Monotherapy arms (oil alone vs. Haridra alone)	combined with oral turmeric capsules		rose; DNA content rose		care control arm
**Dewi *et al.* [54] Diabetic Wounds Controlled *in vivo* animal study**	**Model:** STZ-induced full-thickness excision (5 mm). **Subjects:** Male white rats (N=25). **Comparator:** 0.9% NaCl (neg); Povidone-iodine (pos)	**Material:** Authenticated leaves. **Extraction:** 96% ethanol maceration. **Formulation:** Ointment in virgin coconut oil base (5%, 10%, 15% w/v)	Daily topical application for 14 days	10% formulation optimal; accelerated closure and reduced serum IL-6 by 43.80% ($p<0.05$); 15% did not outperform 10%	**Profiling:** Qualitative tube tests only. **Standardized:** No (crude extract)	Small sample size (n=5/group); non-linear dose-response unresolved; reliance on antiseptic comparator rather than specialized hydrogels; short follow-up
**Lestari *et al.* [55] Diabetic Wounds Comparative *in vivo* animal study**	**Model:** Alloxan-induced excision (1.5 cm). **Subjects:** Male Wistar rats (N=15). **Comparator:** Untreated diabetic control; non-diabetic normal control	**Material:** Local leaves. **Extraction:** 70% ethanol maceration. **Formulation:** Hydrogel wound dressing patch	Topical patch replacement every 3 days; assessed days 0, 7, 14, 21	Wound closure comparable to non-diabetic normal healing ($p>0.05$) over 21 days; facilitated sustained release	**Profiling:** Physical characterization only. **Standardized:** No (crude extract)	Extract concentration omitted; qualitative testing only; small cohort; Alloxan model less representative of human type 2 diabetes than STZ/high-fat models
**Bhagavathy *et al.* [56] Diabetic Wounds *Preclinical in vivo* study**	**Model:** STZ-induced deep partial-thickness thermal burn (70°C, 35s). **Subjects:** Male Swiss albino mice (N=15). **Comparator:** SSD ointment base; commercial SSD 1% cream	**Material:** Local leaves. **Extraction:** Aqueous extraction (biogenic reducing agent). **Formulation:** Green synthesized AgNPs (2.5 & 5 mg) in SSD base	Daily topical application for 25 days; assessed days 5, 10, 15, 20, 25	97.87% wound contraction by day 20; accelerated histological angiogenesis, re-epithelialization, and tissue regeneration	**Profiling:** Nanoparticle characterization (UV-Vis, FTIR, XRD, SEM). **Standardized:** No (extract unquantified)	Plant extract used solely as reducing agent; thermal injury evaluated under diabetic conditions rather than excisional ulceration; very small sample size (n=3)

**Rajora *et al.* [57] Diabetic Wounds *In vivo* biomaterial scaffold study**	**Model:** STZ-induced full-thickness dorsal excision (8 mm). **Subjects:** Male Swiss albino mice (N=18). **Comparator:** Commercial antibiotic ointment; untreated diabetic control	**Material:** Gum polysaccharide (AIG). **Extraction:** Aqueous dissolution and ethanol precipitation. **Formulation:** Nanofibers (AIG 3% + PVA 7% + Curcumin)	Nanofiber mat under sterile secondary gauze; continuous 18-day evaluation	Complete (100%) closure by day 18 ($p=0.0004$); thick re-epithelialization, prominent neovascularization; hydroxyproline elevated (5.1 vs. 1.8 μg/mg)	**Profiling:** Tensile, FTIR, DSC, SEM, release kinetics. **Standardized:** No (structural polymer; no neem limonoids)	Evaluates gum polysaccharide rather than bioactives; composite system prevents isolating neem-specific healing mechanisms from curcumin and PVA
**Mainetti *et al.* [9] Burn Wounds Retrospective pediatric case series**	**Model:** Partial- to full-thickness 2nd-degree burns. **Subjects:** Human pediatric patients (N=9). **Comparator:** None (uncontrolled series)	**Material:** Commercial seed oil. **Extraction:** Commercial cold pressing. **Formulation:** Topical oily solution (*A. indica* + *H. perforatum*, 1:1)	Topical application until complete healing; frequency unstated	Marked pain reduction; complete soft-tissue re-epithelialization without surgical grafting or hypertrophic scars	**Profiling:** None reported. **Standardized:** No (commercial crude oil mixture)	Absence of control group; small sample size (n=9); dose and frequency omitted; impossible to isolate neem bioactivity from St. John's wort
**Anjum *et al.* [59] Burn Wounds Comparative *in vivo* animal study**	**Model:** Contact thermal burn via hot iron plate. **Subjects:** Male New Zealand White rabbits (N=16). **Comparator:** Untreated; commercial Bactrocin®; crude neem alone	**Material:** Local leaves. **Extraction:** Aqueous extraction. **Formulation:** Topical paste (neem extract 4 g + ZnO NPs 1 g)	Applied topically twice daily for 28 days	Significantly accelerated contraction rate and shortened closure time vs. neem alone or Bactrocin ($p<0.05$)	**Profiling:** Nanoparticle characterization (SEM/EDX for ZnO). **Standardized:** No (crude leaf extract)	Small sample size; burn depth not histologically verified; rabbit skin contraction physiology diverges from human burns; independent bioactivity of ZnO core unisolated

**Bhatia *et al.* [60] Burn Wounds Controlled *in vivo* animal study**	**Model:** Scald burn (90°C water for 15s). **Subjects:** Wistar albino rats (N=70). **Comparator:** Normal control; Silver sulfadiazine (1%)	**Material:** Leaves (*A. indica* and *Morus alba*). **Extraction:** Aqueous extraction. **Formulation:** 10% + 10% combination cream	Topical cream applied for 21 days	MA + AI combination cream achieved 93.03% planimetric contraction on day 11 (highest among all arms); enhanced granulation	**Profiling:** Qualitative tube tests only. **Standardized:** No (crude aqueous extracts)	Lacked histological confirmation of full-thickness dermal necrosis; daily application frequency omitted; proposed synergy hypothetical
**Arena *et al.* [42] Infected Wounds Randomized Controlled Trial (Phase III)**	**Model:** Dehisced surgical wounds with critical colonization/infection. **Subjects:** Human patients (N = 99 enrolled, 97 completed). **Comparator:** Silver-based active dressings	**Material:** Neem oil and Hypericum extract. **Formulation:** Primary Wound Dressing [ONE] (1PWD)	Evaluated at baseline and days 7, 14, 21, and 28	Pain reduction significantly favored the hypericum-neem dressing ($p<0.0001$); no statistically significant difference in overall BWAT scores ($p=0.81$)	**Profiling:** Unstated. **Standardized:** Commercial medical device	Co-formulation prevents isolating neem's specific contribution; non-significant BWAT differences do not establish equivalence; lack of quantitative microbiological eradication data
**Bwala *et al.* [62] Infected Wounds Veterinary clinical case report**	**Model:** Post-operative surgical wound (7 cm) with purulent infection. **Subjects:** Domestic ram (*Ovis aries*, n = 1). **Comparator:** None	**Material:** Locally sourced seed kernels. **Extraction:** Traditional crude mechanical pressing. **Formulation:** 100% crude undiluted seed kernel oil	Single topical application on day of surgery; 7-day observation	Complete macroscopic healing of 7 cm infected lesion within 7 days without antibiotics	**Profiling:** None reported. **Standardized:** No (crude unrefined seed oil)	Single veterinary subject; absence of control group; lacking quantitative bacteriological culture or CFU counts; applied volume unquantified; spontaneous healing cannot be ruled out
**Villani *et al.* [63] Infected**	**Model:** Dorsal burn infected	**Material:** Commercial	~100 mg paste applied as	Significantly increased	**Profiling:** Rheology,	Invertebrate model lacking

**Wounds Invertebrate *in vivo* model & *in vitro* assays**	with *P. aeruginosa* and *S. aureus*. **Subjects:** Wax moth larvae (*Galleria mellonella*). **Comparator:** Untreated; plain BC paste; topical oxytetracycline	seed oil. **Extraction:** Cold pressing. **Formulation:** Neem-Hypericum-BC wound care paste	single treatment 1 h post-infection; 4-day survival monitoring	larval survival over 96h post-infection; reduced bacterial bioburden *in vitro*	moisture retention, BC structural analysis. **Standardized:** No (oil unstandardized)	mammalian immune cells and vascularized repair; short follow-up (4 days); tested on laboratory strains rather than clinical isolates
**Sahni & Dubey [52] Incision & Excision Wounds Uncontrolled clinical study**	**Model:** Mixed wounds (including 5 incised wounds and 1 burn). **Subjects:** Human patients (n = 20). **Comparator:** None	**Material:** Leaves, stem/root bark, flowers, fruits. **Formulation:** Oral polyherbal powder and topical neem leaf decoction	Oral powder (3 g twice daily for 30 days) + topical leaf decoction on alternate days	Wound-size severity scores decreased from 1.70 to 0.15 (91.17% improvement); pain and symptom scores improved ($p<0.001$)	**Profiling:** None reported. **Standardized:** No	Absence of control group; mixed etiologies prevent estimation of separate effect for incisions; simultaneous oral and topical treatment prevents isolating therapeutic route
**Chundran *et al.* [43] Incision & Excision Wounds Controlled *in vivo* animal study**	**Model:** Full-thickness dorsal excision (1.5 cm diameter). **Subjects:** Male Wistar rats (n = 20). **Comparator:** 0.9% normal saline (neg); 10% Povidone-iodine (pos)	**Material:** Authenticated local leaves. **Extraction:** Maceration in 96% ethanol for 72h. **Formulation:** Topical leaf extract ointment	Daily topical application for 15 days; assessed on days 0, 5, 10, 15	Significant wound diameter reduction vs. saline ($p<0.01$); healing rate comparable to povidone-iodine; early fibroblast proliferation	**Profiling:** None reported. **Standardized:** No (crude ethanolic extract)	Small sample size; reliance on simple macroscopic planimetry; lack of analytical quantification or HPLC profiling; absence of quantitative histological scoring
**Nagesh *et al.* [66] Incision & Excision Wounds**	**Model:** Full-thickness excision and linear incision	**Material:** Leaves (Himalaya Drug	Excision: once daily until complete closure.	Faster contraction than controls and	**Profiling:** Commercial grade (batch HPLC	Commercial crude extract evaluated without batch-

**Comparative *in vivo* animal study**	wounds. **Subjects:** Wistar albino rats (n = 36). **Comparator:** Simple ointment base; Framycetin sulfate cream	Company). **Extraction:** Industrial ethanolic extraction. **Formulation:** 5% w/w extract in simple ointment base	Incision: once daily for 10 days	framycetin; significantly superior incision breaking tensile strength on day 10 ($p<0.01$)	unstated). **Standardized:** No (marker percentages unquantified)	specific chromatographic fingerprint; intracellular mechanistic signaling pathways unassayed
**Imran *et al.* [67] Incision & Excision Wounds Controlled *in vivo* animal study**	**Model:** Full-thickness dorsal excision (~150 mm²). **Subjects:** Male Wistar rats (n = 18). **Comparator:** Untreated control; simple ointment base	**Material:** Leaves (*A. indica, S. persica*) & flowers (*C. officinalis*). **Extraction:** Ethanolic maceration. **Formulation:** Polyherbal ointment (10% w/w mixed extracts)	Applied topically once daily for 19 days	Complete wound closure achieved by day 19; accelerated scab detachment and dermal re-epithelialization	**Profiling:** Qualitative tube tests only. **Standardized:** No (polyherbal crude mixture)	Uninfected excision model; individual phytochemical contributions of *A. indica* within polyherbal matrix unisolated; qualitative histological assessment only
**Nasrine *et al.* [68] Incision & Excision Wounds *In vivo* biomaterial scaffold study**	**Model:** Full-thickness excision (2 cm diameter). **Subjects:** Male Wistar albino rats (n = 18). **Comparator:** Untreated control; plain silk fibroin hydrogel; plain neem hydrogel	**Material:** Local leaves. **Extraction:** Aqueous extraction. **Formulation:** Neem-silk fibroin hydrogel (N-SFB-HG)	Applied topically; dressing changed every 3 days up to day 21	Accelerated wound contraction by day 21; enhanced structural re-epithelialization; *in vitro* inhibition against *E. coli* and *S. aureus*	**Profiling:** FTIR, XRD, SEM, swelling kinetics. **Standardized:** No (extract unquantified)	Antibacterial activity verified strictly *in vitro*, not challenged against an *in vivo* infected wound bed; tissue repair was scaffold-guided rather than infection-driven
**Rahman *et al.* [69] Incision & Excision Wounds *In vivo* biomaterial study**	**Model:** Full-thickness excision and liver hemorrhage models. **Subjects:**	**Material:** Commercial seed oil. **Extraction:** Mechanical cold pressing. **Formulation:**	Single topical film dressing application; evaluated over 16 days	Rapid hemostasis (91.44% BCI in 5 min); complete closure by day 16 ($p<0.001$);	**Profiling:** FTIR, contact angle, tensile strength. **Standardized:** No (oil	Complex polymeric matrix where physical barrier and fluid handling effects cannot

	Sprague Dawley rats (n = 24). **Comparator:** Plain gauze; blank PVA/chitosan film; commercial patch	Biocomposite film (neem oil in cross-linked PVA/chitosan matrix)		advanced collagen alignment and hair follicle regeneration	unstandardized)	be easily separated from phytochemical bioactivity
**Treichel *et al.* [70] Incision & Excision Wounds Controlled large mammal study**	**Model:** Large thoracic full-thickness excision (7x7 cm). **Subjects:** Adult Santa Inês sheep (n = 8). **Comparator:** 0.9% NaCl saline wash; commercial ointment base	**Material:** Local leaves. **Extraction:** Aqueous infusion and ointment preparation. **Formulation:** Topical aqueous extract wash and topical neem ointment	Daily topical dressing changes for 28 days	Reduced clinical signs of inflammation, but failed to accelerate wound contraction or healing time vs. saline ($p>0.05$)	**Profiling:** None reported. **Standardized:** No (concentrations omitted in paper)	Large-animal null outcome directly contrasts rodent data; applied extract concentrations omitted by authors; lack of analytical standardization
**Olorunsola *et al.* [71] Incision & Excision Wounds Comparative route study**	**Model:** Full-thickness circular excision (1 cm diameter). **Subjects:** Albino rats (n = 20). **Comparator:** Untreated control; vehicle control	**Material:** Air-dried leaves. **Extraction:** Aqueous foliar extraction vs. whole powdered leaves. **Formulation:** Topical aqueous extract vs. oral feed	Daily application vs. daily oral dietary feeding over 14 days	Topical neem showed 92% wound contraction ($p<0.05$) and faster closure than oral feeding; shows topical superiority	**Profiling:** Qualitative tube tests only. **Standardized:** No	Pharmacokinetic parameters (local bioavailability, first-pass metabolism) not directly quantified; small sample size lacks vehicle-only negative control arm
**Iqbal *et al.* [72] Incision & Excision Wounds *Preclinical in vivo* study**	**Model:** Full-thickness excision (2 cm diameter). **Subjects:** Male albino rats (n = 20). **Comparator:** Untreated	**Material:** Local leaves. **Extraction:** Green biosynthesis with zinc and silver salts. **Formulation:** ZnO and Ag-	Topical hydrogel application every 48h over 14 days	Reduced tissue edema; accelerated epithelialization by day 14 via moist wound bed and exudate control	**Profiling:** SEM, TEM, XRD, FTIR, UV-Vis. **Standardized:** No (reducing agents only; unquantified)	Phytochemical contribution indistinguishable from nano-metal and polymeric chitosan bioactivity; wound repair

	control; blank chitosan hydrogel; pure ZnO NPs	ZnO NPs in chitosan hydrogel matrix				driven largely by biomaterial matrix
**Maan *et al.* [73] Incision & Excision Wounds Controlled *in vivo* animal study**	**Model:** Full-thickness excision (177 mm²) and incision models. **Subjects:** Swiss albino mice (n = 30). **Comparator:** Simple ointment base; Povidone-iodine (5% w/w)	**Material:** Authenticated stem bark. **Extraction:** Successive: aqueous, 95% ethanol, hydroethanolic. **Formulation:** 15% w/w bark extract in ointment	30 mg applied topically once daily for 14 days (excision); breaking strength on day 10	Aqueous stem bark extract significantly accelerated contraction, elevated hydroxyproline, and increased incision breaking strength ($p<0.01$)	**Profiling:** Phenolic and flavonoid content. **Standardized:** No (unstandardized for specific tannins)	Rodent loose-skin contraction model differs from human re-epithelialization; lack of purified constituent isolation; superiority of aqueous fraction unassayed analytically
**Babu *et al.* [74] Incision & Excision Wounds Comparative *in vivo* animal study**	**Model:** Full-thickness circular excision (5 mm²). **Subjects:** Albino Wistar rats (n = 24). **Comparator:** Control ointment base; standard Povidone-iodine	**Material:** Local stem bark. **Extraction:** 95% ethanol and cold aqueous maceration. **Formulation:** 10% w/w ethanolic and 5% w/w aqueous bark ointments	Applied topically once daily for 15 days	Ethanolic and aqueous bark ointments promoted faster contraction and epidermal maturation than control and povidone-iodine ($p<0.01$)	**Profiling:** Qualitative tube tests only. **Standardized:** No (crude unstandardized extract)	Benchmarked against antiseptic agent (povidone-iodine) rather than modern regenerative dressings; very small excision wound area (5 mm²); lacks quantitative collagen subtyping
**Ajayi *et al.* [75] Incision & Excision Wounds Controlled *in vivo* animal study**	**Model:** Full-thickness excision wound. **Subjects:** Female Wistar albino rats (n = 30). **Comparator:** Soft yellow paraffin vehicle control	**Material:** Local seeds. **Extraction:** Maceration in polar methanol and non-polar n-hexane. **Formulation:** 5% and 10% v/v seed	Applied topically daily over 21 days	5% methanolic seed ointment produced fastest wound closure ($p<0.05$); non-linear dose-response documented (5% outperformed 10%)	**Profiling:** Qualitative tube tests only. **Standardized:** No (crude extracts without HPLC)	Paradoxical non-linear dose response (5% > 10%) unexplained; possible cytotoxic or pro-inflammatory thresholds at elevated limonoid

		ointments in paraffin				doses unassayed
**Kaur *et al.* [44] Nimbidin (Isolated Bioactive) *In vitro* & *in vivo* acute inflammation**	**Model:** *In vitro* macrophage/neutrophil activation; *in vivo* peritoneal exudation. **Subjects:** Rat peritoneal macrophages & human neutrophils. **Comparator:** Vehicle control; LPS stimulation alone	**Compound:** Nimbidin (purified tetranortriterpenoid fraction). **Isolation:** Chromatographic fractionation from neem seed oil. **Formulation:** *In vitro* culture medium; oral gavage	*In vitro*: 5-100 μg/mL. *In vivo*: 5-25 mg/kg orally	Suppressed macrophage migration, NO production (via reduced iNOS induction), and PGE2 release; inhibited neutrophil degranulation (MPO, β-glucuronidase)	**Profiling:** TLC and UV-Vis validation. **Standardized:** Yes (purified chromatographic fraction)	Evaluated in isolated inflammatory cell cultures; endpoints not evaluated within an actual cutaneous wound bed or mammalian skin injury model
**He *et al.* [45] Azadirachtin (Isolated Bioactive) Controlled *in vivo* flap study**	**Model:** Random skin flap ischemic necrosis. **Subjects:** Sprague Dawley rats (n = 36). **Comparator:** Sham control; vehicle control (saline)	**Compound:** Azadirachtin A (isolated pure standard, purity ≥ 95%). **Isolation:** Commercial analytical reference standard. **Formulation:** Intraperitoneal (IP) systemic injection	100 and 200 mg/kg/day IP for 7 consecutive days	Significantly expanded surviving flap area; upregulated VEGF, CD31, and eNOS; downregulated caspase-3, IL-1β, and TNF-α	**Profiling:** HPLC and mass spectrometry verification. **Standardized:** Yes (chemically authenticated, ≥ 95% purity)	Flap survival model represents ischemic preconditioning rather than standard full-thickness open excisional wound healing; systemic IP injection rather than topical delivery
**Sudhakaran *et al.* [47] Nimbin (Isolated Bioactive) *In vitro* enzymatic & *in vivo* zebrafish**	**Model:** *In vitro* LOX/proteinase assays; *in vivo* H_2O_2 oxidative stress. **Subjects:** Human erythrocytes; zebrafish (*Danio rerio*) embryos.	**Compound:** Nimbin (N1, pure crystalline isolate) & semi-synthetic analogs. **Isolation:** Column chromatography from seeds. **Formulation:** Buffer	*In vitro*: 10-50 μM; *In vivo*: 5-20 μM in tank water	*In vitro* lipoxygenase inhibition (64.1% at 25 μM) and proteinase inhibition; reduced *in vivo* ROS to 63.65% and attenuated lipid peroxidation	**Profiling:** HPLC, 1H-NMR, 13C-NMR, and FTIR spectral confirmation. **Standardized:** Yes	Did not measure TNF-α, IL-1β, or NF-κB nuclear translocation; tested in non-mammalian zebrafish; unvalidated in mammalian cutaneous wound healing

	**Comparator:** Vehicle control; standard anti-inflammatory drugs	solution; aqueous immersion				
**Sim *et al.* [49] Gedunin (Isolated Bioactive) *In vitro* human cell culture study**	**Model:** *In vitro C. acnes*-induced cutaneous inflammatory model. **Subjects:** Human keratinocytes (HaCaT) and fibroblasts (HDF). **Comparator:** Untreated cells; vehicle control; stimulus alone (*C. acnes*)	**Compound:** Gedunin (pure limonoid isolate, purity > 98%). **Isolation:** Commercial analytical reference isolate. **Formulation:** Cell culture medium with DMSO vehicle	1-10 μM *in vitro* culture concentrations	Inhibited IκBα phosphorylation/degradation; blocked NF-κB p65 nuclear translocation; reduced pro-inflammatory mediators; increased collagen by 136.8% in fibroblasts	**Profiling:** HPLC and MS (>98% purity). **Standardized:** Yes (pure reference limonoid isolate)	Conducted entirely in cell culture (*in vitro*); *in vivo* pharmacodynamics and tissue regeneration unverified in animal wound models
